# Investigation of Magnesium Hydroxide as a Halogen-Free Fire-Retardant Filler for Advanced Polymer-Based Solutions: A Review

**DOI:** 10.3390/polym18111386

**Published:** 2026-06-03

**Authors:** Federico Ferrante, Giuseppe Battaglia, Giorgio Micale, Nadka Tz. Dintcheva

**Affiliations:** Dipartimento di Ingegneria, Università di Palermo, Viale delle Scienze, ed. 6, 90128 Palermo, Italy; federico.ferrante01@community.unipa.it (F.F.); giuseppe.battaglia03@unipa.it (G.B.); giorgiod.maria.micale@unipa.it (G.M.)

**Keywords:** flame retardants, magnesium hydroxide, bitterns, seawater, composites, polymers, biopolymers, halogen-free

## Abstract

Magnesium hydroxide is attracting growing interest as a versatile, halogen-free flame retardant, and this review surveys its production routes, structure–property relationships and use in polymer systems from commodity polyolefins to advanced bio-based materials. Industrial Mg(OH)_2_ is still predominantly obtained from mining or hydration of MgO, but increasing attention is being devoted to recovery from seawater and saltwork brines, where precipitation from Mg^2+^-rich streams followed by controlled rehydration or direct precipitation yields fine, high-purity powders suitable for flame retardant use and simultaneously valorizes saline wastes. In parallel, hydrothermal synthesis has been extensively explored to tailor particle size and morphology by adjusting the precursor, solvent, temperature and time, enabling high-surface-area Mg(OH)_2_ or MgO with narrow size distributions that are attractive for high-performance composites also evaluated via ball milling, crushing and refining. More recently, process intensification strategies such as microwaves and ultrasounds have been proposed to shorten reaction times, lower temperatures and better control nucleation and growth, opening paths toward energy efficient production of structured Mg(OH)_2_ from both conventional and brine-derived precursors. The second part of the review analyzes how the intrinsic endothermic decomposition and basic character of Mg(OH)_2_ can be utilized across a broad range of polymer matrices and how surface functionalization strategies extend its applicability. In addition to “as received” powders, stearic acid and other fatty acids, metal soaps and various organic coupling agents are widely used to render the surface more hydrophobic, enhance dispersion and interfacial adhesion, and in some cases introduce additional char-forming or barrier functionality. In terms of the application, the review methodically synthesizes and contrasts fire and mechanical data for Mg(OH)_2_-containing polyolefins (HDPE, LLDPE, PP and EVA) utilized in cables and building products, expandable polymers and foams, biopolymers (PLA and PBS), and elastomers. The review places particular emphasis on the balance between loading level, processability, flame performance and mechanical integrity. This review aims to provide a comprehensive framework for designing next-generation Mg(OH)_2_-based flame-retardant systems for both conventional and emerging polymer technologies. To this end, it integrates advances in sustainable feedstocks, controlled synthesis and surface engineering with the rapidly expanding application space.

## 1. Introduction

**Figure 1:**
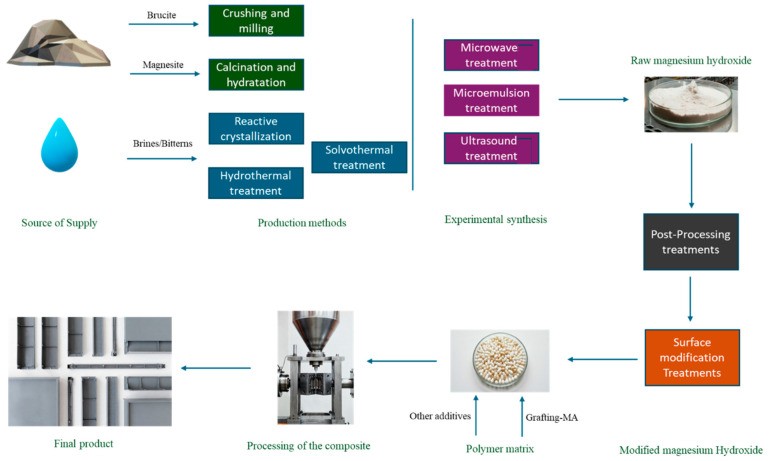
Visual summary of the production and the industrial application of magnesium di-hydroxide (MDH).

In nature, magnesium di-hydroxide (chemical formula: Mg(OH)_2_) is best known as the mineral brucite, with notable deposits in the Ural Mountains, California, Italy and Greece. The compound is sparingly soluble in water, moderately basic, and is widely used in non-polymer applications such as antacids and wastewater neutralization because of its ability to buffer acidic media. The key property of magnesium hydroxide is its endothermic thermal decomposition, in which Mg(OH)_2_ converts to MgO and water vapor, absorbing a substantial amount of heat and diluting combustible gases. These features endow Mg(OH)_2_ with dual functionality as both a heat sink and a source of inert gases in polymer matrices exposed to fire, in contrast to halogenated flame retardants that primarily act through gas-phase radical trapping. The systematic investigation of magnesium hydroxide and related metal hydroxides as thermal stabilizers and flame retardants for PVC and polyolefins commenced in the late 20th century, driven by the need to control HCl evolution, reduce smoke and replace antimony, chlorine and bromine-based additives in numerous applications. The growing regulatory pressure and legislation violations to ban halogen-based flame retardants in favor of halogen-free systems has made magnesium hydroxide an attractive candidate for polymer applications. At the same time, the cost and environmental footprint of Mg(OH)_2_ depend critically on how it is produced, which unify the importance of developing cost-effective and sustainable routes such as direct precipitation from seawater and saltwork brines, hydrothermal crystallization, brucite crushing and milling or even innovative and more controlled routes like microwave- or ultrasound-assisted synthesis that reduce energy demand and enable fine morphology control. As described in Figure 1, the process begins with the raw material source and the process to obtain raw MDH, driven by the obtainment of the correct particle size and morphological structure that should be very well defined during the precipitation or post-production sections of the process because of the standards that the market requires to be considered both a sustainable and performative additive for innovative stable composites. With that said, it is well known that incorporating magnesium hydroxide into polymer matrices is therefore worth detailed investigation not only because it can provide efficient, halogen-free flame retardancy, but also because its layered structure and basic surface chemistry make it highly tunable through surface modification. Stearic acid, other fatty acids, metal soaps and silane-based treatments are commonly employed to render Mg(OH)_2_ more organophilic, improve dispersion and interfacial adhesion, and, in some cases, introduce additional char-forming or barrier-enhancing functionalities, while keeping high, or at least acceptable, mechanical properties for the processing and production of the final product. This versatility allows Mg(OH)_2_ to be tailored for a wide spectrum of matrices, including expandable polymers and foams, polyolefins, biopolymers and elastomers, where it can be used alone or synergistically with other fillers to enhance fire safety while maintaining the required mechanical and processing properties. As a result, magnesium hydroxide stands out for safer and more environmentally benign flame-retardant technologies, expanding the market of polymeric products in everyday life and in critical sectors such as construction, transportation and electronics to become both more fire-resistant and more sustainable.

## 2. Production of Magnesium Di-Hydroxide (MDH)

Magnesium Di-Hydroxide (MDH), whose mineral name is brucite as already discussed in the introduction, is a sparingly soluble inorganic compound (0.009 g L^−1^ at 18 °C in water) that crystallizes where Mg^2+^ occupies octahedral sites coordinated by six ligands (hydrogen) arranged in neutral layers stabilized by hydrogen bonding. This layered crystal lattice emphasizes the platelet-like or flake-like morphologies with the possibility of anisotropic growth and a frequent tendency to form aggregates and agglomerates, which can be considered as a critical aspect during the synthesis; it is necessary to carefully manage this phenomenon and reduce it as much as possible to prevent the incompatibility with the polymer matrix during the processing of the final product [1,2]. The following paragraphs will critically analyze MDH synthesis processes with a special focus on concerns of their scale-up and feasibility. Special attention will be given to extraction from brines and waste waters in accordance with the principles of circular economy and environmental sustainability.

Magnesium hydroxide supply can be obtained from a wide spectrum of natural and anthropogenic sources, and this plurality of feedstocks has an impact on its availability, purity, costs, structure and sustainability for specific applications, including brucite, magnesite, carbonated dolomite and skarn deposits [3,4]. MDH is found predominantly in the mineral brucite and its industrial exploitation typically involves quarrying and open-pit mining, followed by primary crushing, grinding and often followed by reprecipitation in water to obtain a brucite-rich fraction or pure MDH for flame retardant applications [5,6,7]. Depending on the end-of-use specification, flotation or selective milling are also used to reduce impurities like calcite, dolomite, silicates and iron-bearing phases. Furthermore, in some industrial chains, brucite ore is intentionally calcined to magnesium oxide (MgO) and then rehydrated under controlled conditions to produce MDH with tailored particle morphology and surface properties, effectively decoupling geological texture from final product characteristics while still relying on the mineral resource as the primary magnesium carrier [8]. Recently, commercially relevant magnesium hydroxide is produced via synthetic precipitation from aqueous streams like seawater, inland brines and process liquors. All these feedstocks contain a rich amount of magnesium as Mg^2+^ and the aim of the process is to induce its precipitation; the process flow also includes separation of the produced MDH by solid–liquid separation steps such as sedimentation, flotation or filtration followed by washing downstream processes to remove soluble salts such as chlorides or sulfates or even residual reagents if needed. The washed cake is then dried and ready to be milled to the desired particle size, producing a technical-grade MDH that can be used directly as a polymer filler for flame retardancy purposes, or as an acidic neutralized agent or intermediate for MgO production for refractory or building applications [9].

This network of different options defines a broad and flexible landscape for MDH supply, within which specific recovery and processing routes can be tailored according to local resource availability, target applications and sustainability criteria.

### 2.1. Commercial Production of Mg(OH)_2_

At the state-of-the-art level, to explain how MDH is produced, it is important to investigate the commercial production of magnesium hydroxide for flame retardant applications. Industrial routes start with magnesia or magnesium chloride solutions and then combine controlled hydration with high-energy drying and milling to deliver powders with tightly specified particle size, porosity and rheological behavior. The emphasis in these processes is not only on generating chemically pure Mg(OH)_2_, but also on engineering surface area, pore structure and agglomeration state so that the filler can be used at high loadings in polyolefins, EVA, PVC and elastomeric matrices without unacceptable increases in viscosity or loss of processability.

**Figure 2:**
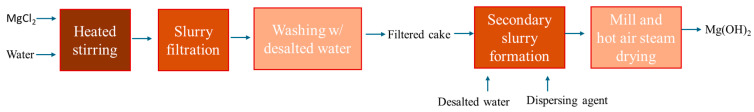
Block flow diagram of Magnifin process.

In the Magnifin patent, visually described in Figure 2, the core concept is to start from a slurry of magnesium hydroxide and then use “mill drying” to simultaneously dry and micronize the particles, followed by a dedicated deagglomeration step. The Mg(OH)_2_ slurry itself is obtained by hydrating a magnesium oxide suspension: MgO is typically produced by spray-roasting a purified MgCl_2_ solution, which is then suspended in water and reacted at 50–100 °C under continuous stirring until hydration to brucite is complete. After hydration, the first slurry is filtered and washed with desalted water to reduce dissolved impurities, yielding a filter cake that is re-slurried with either water or a dispersing agent (polyacrylates, organic acids, naphthalene sulfonate condensates, nonionic polyether surfactants, etc.) to produce a second slurry with 20–40 wt% Mg(OH)_2_ in water or up to 80 wt% when dispersants are used. This high-solids slurry is then fed to a mill drying unit in which a rapidly rotating rotor and a high-throughput hot air stream generate intense turbulence, lifting the slurry into the air vortices and drying droplets in flight; the result is a mill-dried Mg(OH)_2_ powder with a BET surface area increased by about 10–40% versus the starting slurry particles. Subsequent separation of the dried product from the air stream with filters yields a powder that already shows significantly lower oil absorption and smaller median pore radius than conventionally spray-dried grades, but which still contains soft agglomerates that may compromise compounding performance. To address this, the same patent specifies a deagglomeration stage in which the mill-dried powder is processed in dry or wet pin mills and/or air classifiers that selectively break agglomerates while preserving the primary particle size (d_50_ of the final product ≥90–95% of the d_50_ of the mill-dried feed). Classification is tuned so that the magnesium hydroxide “product particles” meet narrow specifications: d_50_ below about 3.5 μm (with preferred sub-ranges down to ≈0.3–1.3 μm), BET-specific surface area between 1 and 15 m^2^ g^−1^ (with distinct product lines in the 1–5, 3–7, 6–10 and 8–12 m^2^ g^−1^ windows), and median pore radii r_50_ in the 0.01–0.5 μm range as determined by mercury porosimetry. The patent further correlates these structural parameters with linseed oil absorption, defining families of products with oil absorption values of roughly 15–40 g per 100 g, which directly influence wetting, dispersion and compound viscosity. Comparative examples show that mill-dried and deagglomerated products exhibit about 30% higher BET, ≈20% smaller median pore radius and ≈24% lower oil absorption than spray-dried Mg(OH)_2_ made from the same slurry, and that these differences translate into reduced torque and energy fluctuations in extruders, higher throughput, lower melt viscosity and improved mechanical properties in EVA/LLDPE compounds [10].

**Figure 3:**
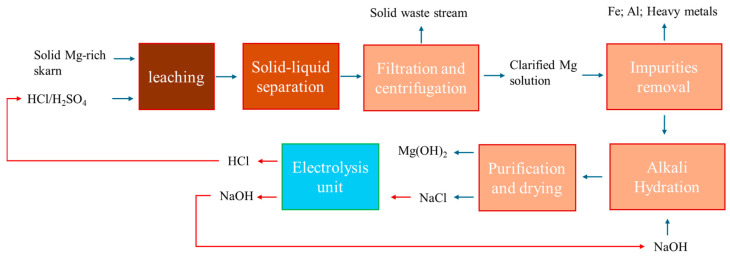
Block flow diagram of the acid leaching/alkaline precipitation process.

Another interesting route is acid leaching/alkali precipitation which targets high-purity Mg(OH)_2_ from mineral or industrial feedstocks. In the flowsheets depicted in Figure 3, magnesium is first transferred into solution by leaching a solid raw material with a mineral acid, followed by purification and precipitation with an alkali compound. The leaching section typically consists of a cascade of stirred tank reactors or leach columns, where the comminuted starting material is contacted with an aqueous acid like HCl or H_2_SO_4_ at elevated temperature and controlled solid to liquid ratio. The aim is to dissolve magnesium, forming MgCl_2_ or MgSO_4_, while leaving silicate gangue and other inert phases in the solid residue; when carbonates are present, CO_2_ is released and managed as a separate gas stream. The resulting slurry is then subjected to solid–liquid separation (thickening, filtration or centrifugation), producing a clarified magnesium solution and a solid waste stream containing insoluble inert substances and carbonate decomposition products. This separation step is critical because it determines the impurity burden entering the precipitation stage; additional impurity removal, such as pH-controlled precipitation of Fe, Al or heavy metals, may be interposed to reach the desired purity level. In the precipitation section, the purified Mg salt solution is fed to a reactor where it is contacted with an alkali, such as NaOH or KOH, to form a suspension of Mg(OH)_2_. This reaction is conducted under conditions that ensure efficient mixing and rapid neutralization, generating a Mg(OH)_2_ suspension that is then fed to a solid–liquid separation step. Separation can be achieved by filtration, sedimentation or centrifugation, yielding high-purity Mg(OH)_2_ as a wet cake and a liquor containing dissolved sodium or potassium salts. The washed Mg(OH)_2_ cake is then dried and, if necessary, further processed (for instance calcined and rehydrated, milled, and classified) to tailor its physicochemical properties for specific applications. A distinctive feature of this layout is the integration of an electrochemical regeneration unit, which converts the liquor salts back into acid and base, thereby closing the reagent loop. For example, NaCl originating from the neutralization step can be fed to an electrolysis unit that produces HCl and NaOH; the HCl is recycled to the leaching section, while NaOH is recycled to the precipitation section, reducing overall chemical consumption and waste generation [11].

**Figure 4:**
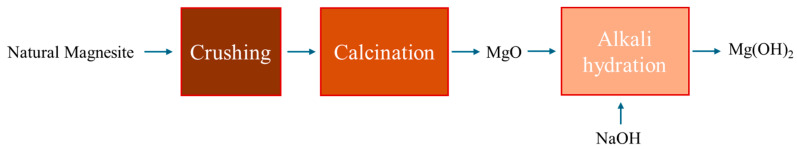
Block flow diagram of the MDH recovery from magnesite route.

The production of magnesium hydroxide from magnesite via calcination–hydration has been refined in several patents, among which EP599085B1 stands out for its detailed description of an industrial “active magnesium hydroxide” process (Figure 4) starting from light-burned magnesia. In this route, natural magnesite is first crushed and calcined under carefully controlled conditions to obtain a reactive MgO called light-burned magnesia with high specific surface area and residual lattice defects, which serves as a suitable precursor for subsequent hydration. The calcined material is then subjected either to wet pulverization followed by hydration in an alkaline aqueous medium at pH ≥ 11 and temperatures typically in the range 85–120 °C, or to a combined hydration and grinding in slurry at temperatures of at least 70 °C. In the first variant, light-burned magnesia is dispersed in water, the pH is adjusted to strongly alkaline values by the addition of alkali, and the suspension is maintained under vigorous agitation and elevated temperature for a defined residence time; under these conditions, MgO is rapidly converted to Mg(OH)_2_, and the simultaneous mechanical dispersion suppresses the formation of coarse agglomerates, yielding a fine, highly reactive hydroxide. In the second variant, MgO powder, water and grinding media are charged to a mill reactor (for example a ball mill) operated at temperatures above 70 °C, so that hydration and comminution proceed concurrently and the growing Mg(OH)_2_ crystallites are continuously broken and deagglomerated, leading to a narrow particle size distribution and a high BET surface area, both of which are key descriptors of “active” magnesium hydroxide in this patent. After completion of hydration, the slurry is optionally classified, dewatered and then dried and milled to obtain a free-flowing powder whose reactivity and uniformity are significantly higher than those of products obtained by simple, low-temperature slaking of MgO, making this process particularly suitable for applications that demand consistent reactivity, such as environmental treatments and high-performance flame-retardant fillers [12].

**Figure 5:**
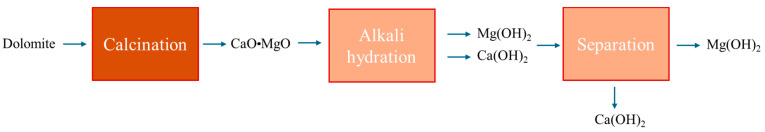
Block flow diagram of mdh recovery from dolomite.

Furthermore, dolomite-based hydration routes exploit the abundance of dolomite and similar rocks by combining thermal decomposition with selective hydration and, in some variants, causticization and carbonation steps. In a simple configuration described in Figure 5, dolomite is first calcined to produce a mixed oxide (CaO·MgO), which is then hydrated under conditions that favor the rapid conversion of MgO to Mg(OH)_2_ while also forming Ca(OH)_2_. The hydration step is carried out in stirred reactors, with controlled water addition and temperature, producing a slurry containing both hydroxides. Depending on the process objective, Ca(OH)_2_ may be separated as a co-product or used in situ. In some layouts, the mixed hydroxide slurry is contacted with a soluble magnesium salt solution (such as MgCl_2_ brine), leading to causticization reactions in which Ca(OH)_2_ reacts with Mg^2+^ to generate additional Mg(OH)_2_ and soluble Ca salts; this increases the overall Mg(OH)_2_ yield and can integrate with brine-based flowsheets. The Mg(OH)_2_-rich phase is then thickened, washed to remove soluble Ca and other ions, and filtered to form a cake that is dried and milled. In more complex schemes, carbonation steps may be included to selectively precipitate CaCO_3_ and leave Mg in solution or in hydroxide form, enabling finer control of Ca/Mg separation and final product purity. Industrial implementations of dolomite routes can be tightly coupled to existing lime and cement plants, where filtration/drying infrastructure already exist [13].

### 2.2. Recovery from Saltwork Bittern

The concept of precipitating magnesium hydroxide from aqueous media has been extended in recent years to more complex and concentrated streams, such as desalination brines, evaporated bitterns and various industrial effluents. Desalination plants using reverse osmosis and thermal processes generate a large volume of brines with elevated Mg^2+^ concentration; instead of disposing of these streams, targeted recovery schemes use alkaline reagents to selectively precipitate Mg(OH)_2_. These flowsheets often include multistage pH adjustments and intermediate separation steps to optimize selectivity and purity, as well as integration with desalination plant operation to minimize chemical and energy consumption and manage the overall water balance [9,14].

**Figure 6:**
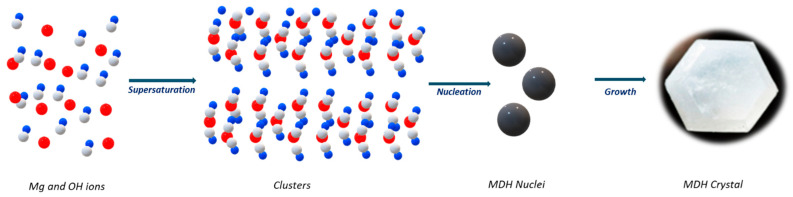
Precipitation mechanism of MDH (i.e., red colored ions are Mg^2+^; white colored atoms—oxygen and blue colored atoms are hydrogen).

The reaction used in this method is called “reactive crystallization” which has emerged as a promising route for MDH production, owing to its intrinsic simplicity, short reaction time and scale-up theoretic feasibility. In this process, an aqueous magnesium source is put in contact with an alkaline solution under conditions that rapidly generate supersaturation, promoting nucleation in the liquid phase. By adjusting operating parameters such as mixing intensity, reactant addition strategy and temperature, reactive crystallization enables efficient conversion of dissolved magnesium into solid MDH. At the same time, the strong coupling between hydrodynamics and kinetics critically influences particle size, aggregation state and morphology, often requiring careful process optimization or complementary post-treatments to obtain a solid in the form of powder with controlled properties demanded by specific applications. The overall reaction can be written as:

$$MgX+2NaOH\;\to Mg{\left(OH\right)}_{2}+\;2NaX$$

where X is the counter ion of magnesium, most commonly chloride in the case of saltwork bitterns. Because the solubility product of magnesium hydroxide is very low, the equilibrium solubility in pure water is only on the order of a few milligrams per liter, in stark contrast to highly soluble salts such as sodium chloride with hundreds of grams per liter at the same temperature. This extremely low solubility means that even modest additions of hydroxide rapidly drive the solution into a strongly supersaturated state, so that Mg(OH)_2_ precipitates almost instantaneously [2,15,16]. As in other reactive crystallization systems, the key elementary steps include formation of a supersaturated solution, primary nucleation, crystal growth and, frequently, aggregation and breakage as expressed in Figure 6, but for Mg(OH)_2_ the kinetics of nucleation and early growth are particularly fast, making supersaturation control central to product design. If hydroxide is added too rapidly, or mixing is insufficient, very high local supersaturation levels are generated around the feed points, leading to intense homogeneous nucleation and the formation of extremely fine, colloidal particles that give rise to the characteristic milky “milk of magnesia” appearance of fresh suspensions. Experimental and theoretical studies have highlighted that the interfacial Gibbs free energy associated with Mg(OH)_2_ clusters in water is relatively low, so that the critical nucleus size lies close to molecular dimensions; under strongly supersaturated conditions, the nucleation barrier is therefore small and a large number of nanoscale nuclei form before any significant growth can occur. The resulting population of nanometric particles exhibits pronounced colloidal behavior, which manifests macroscopically as highly turbid, bluish-white dispersions and can severely impair sedimentation and filtration unless supersaturation, mixing and seeding strategies are carefully optimized [2].

In multi-component brines such as RO concentrates or saltwork bitterns, the ideal equilibrium is further complicated by the presence of competing cations and complexing ligands. Divalent ions like Ca^2+^ consume OH- and can form their own hydroxide or also carbonate and sulphate phases like Ca(OH)_2_, CaCO_3_, CaSO_4_·2H_2_O which co-precipitate with MDH and either incorporate into its lattice or absorb onto its particles, reducing the purity and altering surface charge and aggregation behavior. At the same time, chloride and other ligands may stabilize Mg ions in solution through complex species such as MgCl^+^ or, in the presence of ammonia, Mg(NH3)n^2+^, effectively shifting the precipitation boundary to higher OH^−^ activities and influencing both nucleation rates and crystal habit. As a result, the critical pH for efficient Mg(OH)_2_ precipitation is not a fixed value but depends on total Mg concentration, background electrolyte composition, the presence of complexing agents, and temperature [17,18,19,20]. The solid phase produced is structurally brucite, giving MDH am hexagonal crystal symmetry and a natural tendency to form plate-like particles. Foreign ions, as said, can partially inhibit growth in selected directions and yield thinner, high-surface-area platelets or more equant particles; these morphological changes have direct consequences on filtration, compaction and downstream processing [21].

Reverse osmosis (RO) desalination plants generate hypersaline concentrate streams which remain largely underused despite their substantial mineral content. Seawater typically exhibits total dissolved solids TDS of 35 g/L with a concentration of Mg ions of around 1250 mg/L [22,23]. In RO brines the Mg ion content could be more than doubled, and the same with other divalent ions such as Ca^2+^, along with minor components including K^+^, Sr^2+^ and other metals. The management of these effluents has emerged as a critical environmental concern due to their potential to increase salinity, density, and thermal stratification in receiving marine ecosystems. However, their elevated magnesium content makes them promising feedstocks for resource recovery processes aligned with circular economy strategies [24]. Solar saltwork bitterns are residual liquors remaining after the sequential precipitation of NaCl and, in some cases, CaSO_4_, in solar ponds from seawater. These solutions are significantly enriched in magnesium. These concentrated matrices, exemplified by the long-operating saltworks of Trapani (Italy), inherently favor selective magnesium extraction due to their high Mg^2+^/Ca^2+^ ratios. The bitterns used in the Trapani studies are collected from the final concentration ponds of the Margi saltwork and exhibit Mg^2+^ concentrations up to roughly 80 g/L, with comparatively low levels of Ca^2+^, bicarbonate and transition metals, a composition that is highly favorable to selective Mg(OH)_2_ precipitation with limited co-precipitation of Ca phases.

**Figure 7:**
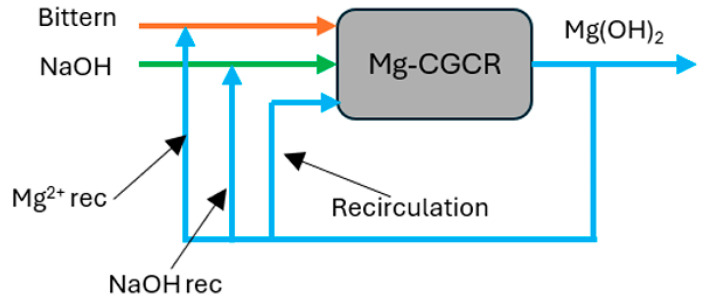
Pilot-scale production of MDH via reactive crystallization reactor strategy in RO industrial plant [29].

At a laboratory scale, Battaglia and colleagues investigated Mg(OH)_2_ recovery from Trapani bitterns by performing batch and continuous experiments in stirred reactors, using synthetic NaOH solutions at stoichiometric and over-stoichiometric doses to achieve final pH values from near neutral to above 12. They showed that when NaOH is dosed to reach strongly alkaline conditions (final pH around 12.5–12.8), magnesium removal efficiencies above 99% can be obtained, essentially achieving quantitative Mg recovery, while maintaining cationic purity of the precipitated solids above 99% and total mass purity above 95%, which aligns with common specifications for commercial Mg(OH)_2_ powders. X-ray diffraction and chemical analyses confirmed that brucite is the dominant phase, with only minor amounts of co-precipitated salts such as NaCl or mixed Mg–Na phases, and that boron, often a critical impurity in bittern-derived products, can be kept below market limits by combining high pH operation with appropriate washing sequences. These laboratory studies also highlighted the sensitivity of particle size distribution and filterability to supersaturation and pH: high pH and fast NaOH addition favor fine, colloidal particles with slower sedimentation and more difficult filtration, whereas controlled addition and seeding promote larger, more compact crystals [25,26]. Building on these results, a pilot scale Magnesium Crystals Granulometry Controlled Reactor (Mg CGCR) was installed and operated at the Trapani saltworks as part of the SEArcularMINE/REWAISE initiatives, whose plant scheme can be observed in Figure 7, providing proof of concept for continuous Mg(OH)_2_ production directly on site. In this configuration, real Margi bitterns are continuously fed to the Mg CGCR together with NaOH solutions of different concentrations, and the reactor is operated under steady-state conditions with well defined residence times; a distinctive feature of the design is the intentional recycling of a fraction of the Mg(OH)_2_ product slurry to the reactor inlet, which serves as a seeding mechanism to moderate supersaturation and to control crystal growth and aggregation. Systematic campaigns were carried out by varying key operating parameters such as bittern flow rate, NaOH concentration, final suspension pH and product recycle ratio, and monitoring their impact on Mg^2+^ extraction performance, solid purity, particle size distribution and dewatering behavior. The pilot scale results confirmed that magnesium recoveries greater than 99% can be consistently achieved at final pH around 12.8, with cationic purity of the Mg(OH)_2_ products always above 99% and overall Mg(OH)_2_ mass purity generally exceeding 95%, even when operating under different NaOH strengths and flow conditions [26,27,28,29].

From a process engineering perspective, continuous crystallization offers several advantages compared to batch stable steady operation, including more consistent production and easier scale-up considerations; these features are especially relevant when the goal is to valorize large volumes of brine for industrial use. Additionally, more advanced reactor concepts have been developed, for instance: a membrane-assisted crystallizer where hydroxide ions migrate through an anion exchange membrane into the brine compartment, inducing MDH precipitation without direct mixing of alkaline reagent and brine. This architecture reduces the risk of co-precipitation and allows employment of lower-cost or less aggressive reagents while still achieving a magnesium recovery efficiency of nearly 100% with a 94–98.8% purity of the recovered mineral [30,31]. Also, it is extremely important to evaluate the process economics and sustainability: efficient precipitation with high yield and purity reduces reagent consumption, minimizes wastes and residuals and improves overall resource recovery, essential for a viable circular economy approach. Continuous processes (especially plug flow or membrane crystallization) enable stable long-term operation, easier integration with existing desalination or saltwork infrastructure, and better control for industrial exploitation. The variability of feed brine composition (RO brine vs. saltwork bittern, differing ion ratios, presence of trace contaminants) demands tailored process design and flexible operational strategies [18]. Given these complexities, as cited before, the literature increasingly emphasizes combined experimental–theoretical approaches—pilot-scale continuous reactors, coupled with the modeling of hydrodynamics, supersaturation kinetics, population balance, and solids separation—to optimize process parameters (mixing, pH, flow rates), maximize magnesium recovery and product purity, and assess feasibility for industrial deployment [25].

**Figure 8:**
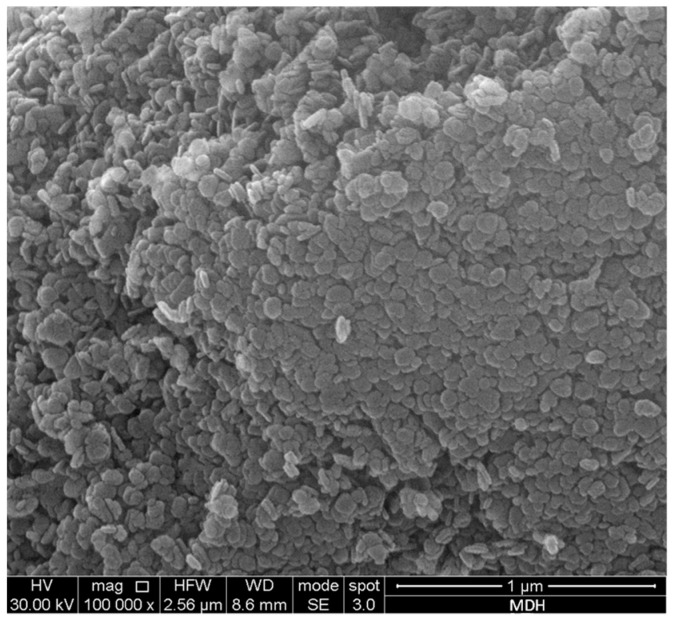
SEM image (an authentic image) of MDH recovered from brine by Trapani (Sicily, Italy).

In the context of Mg(OH)_2_ recovered from reactive precipitation, the formation of large aggregates and hard agglomerates is actually disadvantageous when the target is a powder comparable to commercial flame-retardant grades for polymers. Commercial Mg(OH)_2_ fillers, such as those described in EP0568488, are designed to have tightly controlled primary particle sizes (for example D_50_ ≈ 0.5–1.5 µm) and specific surface areas in the range 13–30 m^2^/g, with minimal presence of agglomerates above 50–100 µm, because oversized granules and strongly bound clusters severely impair dispersion in the polymer melt and lead to defects and poor mechanical properties. In contrast, Mg(OH)_2_ obtained by straightforward precipitation from concentrated solutions (including bitterns) naturally evolves towards broad particle size distributions dominated by aggregated and agglomerated structures as shown in the SEM images in Figure 8. This means that the “natural” outcome of reactive Mg(OH)_2_ precipitation, fast nucleation followed by uncontrolled aggregation, is fundamentally at odds with the microstructural requirements of polymer-grade flame retardants, where primary particle size, shape and surface area must be decoupled from large-scale agglomeration. Population balance and mixing studies show that the operating windows that maximize magnesium recovery from concentrated solutions also promote the formation of fine, highly hydrated agglomerates and compressible cakes, which are unacceptable intermediates if the ultimate goal is a powder that can compete with engineered commercial products. In other words, for flame retardant applications the presence of aggregates and agglomerates is a core obstacle; unless additional unit operations (controlled milling, classification, surface modification) or radically different crystallization strategies are introduced, Mg(OH)_2_ recovered from reactive precipitation will remain theoretically morphologically inferior to purpose-designed commercial fillers, despite its attractive purity [26].

For the sake of comparison, Table 1 summarizes the product prices, productivity, scale-up complexity and final product qualities of MDH powders from the commercial processes discussed thus far.

The following sub-sections provide additional insights into different methods for MDH production, mainly related to studies having academic relevance and/or partially already implemented in industrial schemes, as discussed before.

### 2.3. Hydrothermal and Solvothermal Methods

The term “hydrothermal” is generally used for heterogeneous reactions that take place in water at temperatures above ambient and pressures above 1 atm in a closed vessel. In most cases the solvent is water, and the reactions are carried out in sealed autoclaves or hydrothermal reactors that allow the system to heat above the normal boiling point of water while the pressure rises autogenously with the temperature. Under these conditions, solids that would be practically inert or only sparingly soluble in water at room temperature can dissolve, transform and recrystallize, so hydrothermal processing is widely used to modify phases, morphologies and textures that are inaccessible under standard conditions. Operational windows for hydrothermal treatments are broad and depend strongly on the specific application. At the lower end, many syntheses and transformations are conducted at temperatures just above 100 °C and moderate pressures only slightly above saturation, typically a few bars, where water remains in the liquid state and enhanced ion mobility accelerates dissolution and recrystallization. At the other extreme, some processes operate close to or beyond the critical point of water, at temperatures above about 374 °C and pressures above 22.1 MPa, often referred to as subcritical, near critical or supercritical regimes depending on how closely they approach or exceed the critical conditions. In these high-temperature/high-pressure domains, water properties such as density, dielectric constant and ion product change dramatically, which in turn alters solubility, acid–base equilibria and reaction pathways, enabling rapid dissolution and controlled crystallization of otherwise refractory materials [32,33]. As said, reactive crystallization represents a fast and scalable precipitation method; this approach is highly efficient in terms of conversion and throughput, but often leads to very fine, highly aggregated particles with broad size distributions and poorly controlled morphology, which can be detrimental for applications requiring well defined particle shape and controlled surface properties. To overcome these limitations, a subsequent hydrothermal treatment can be introduced as a post-precipitation maturation step, in which the as-precipitated Mg(OH)_2_ is exposed to elevated temperature and pressure in aqueous medium. Under these conditions, dissolution–recrystallization processes promote crystal reshaping and growth into more regular plate-like hexagonal particles, reducing aggregation and improving crystallinity, thus tailoring the final material characteristics to meet stringent application-specific requirements [34]. Hydrothermal Mg(OH)_2_ synthesis is classically carried out in water using sealed, Teflon-lined stainless steel autoclaves at temperatures between about 120 and 220 °C and residence times from 2 to 24 h [33]. Typical routes start from soluble magnesium salts (MgCl_2_, Mg(NO_3_)_2_, MgSO_4_) or from amorphous/poorly crystalline Mg(OH)_2_ or MgO produced by co-precipitation, which are dispersed in alkaline solution (NaOH, NH_4_OH, hydrazine containing media, etc.) and then hydrothermally treated to yield well crystallized brucite. Under hydrothermal conditions, less stable or highly defective precursor particles partially dissolve; supersaturated solution then feeds the nucleation and growth of new brucite crystallites, so that the overall pathway is best described as dissolution–reprecipitation with concurrent Ostwald ripening. Crystal growth occurs through the stacking of octahedral layers along the c axis, and the relative growth rates on the basal and prismatic faces define the eventual plate-like, wire-like, or rod-like morphologies [35,36,37].

From a process point of view, simple “one pot” hydrothermal syntheses mix the Mg precursor at room temperature, then load the slurry into an autoclave filled to ~60–80% of its volume and heat it to 150–200 °C for 6–18 h, producing brucite nanoflakes, nanowires, or micro-flowers depending on the medium and additives [37,38,39]. For instance, treatment of a Mg(NO_3_)_2_/hydrazine-derived nanocrystalline Mg(OH)_2_ precursor at 160–200 °C led to the systematic growth of hexagonal flakes whose thickness, lateral size, and agglomeration decreased or increased with temperature and time, while crystallinity and lattice ordering improved markedly with prolonged hydrothermal aging [38,40]. In another study, Mg(OH)_2_ and MgO nanostructures were obtained from MgSO_4_, ethylenediamine and hydrazine via a hydrothermal step, with the composition of the solution exerting strong control over particle size and morphology [41]. Process conditions such as temperature, duration, precursor concentration, and autoclave filling ratio also influence nucleation density and ripening, providing handles to tune specific surface area and pore structure for downstream calcination to MgO [36,38,39].

The hydrothermal route is particularly attractive for upgrading low-quality Mg(OH)_2_ obtained from brines; an initial precipitation step produces fine, partially amorphous or defect-rich Mg(OH)_2_, which is then hydrothermally treated to “heal” structural defects, grow platelets, and narrow the size distribution, improving filtration, mechanical behavior, and thermal decomposition characteristics [27,36,42,43]. This strategy, already proposed in the context of brine-derived magnesium compounds, decouples impurity removal (primarily addressed in the wet chemical step) from textural and morphological optimization (concentrating in the hydrothermal stage) [15,44,45]. Plate-like or lamellar Mg(OH)_2_ particles with controlled thickness are attractive as flame-retardant fillers and reinforcing agents in polymer composites, where high aspect ratio and good dispersion are desired, while wire-like or flower-like morphologies can serve as precursors to high-surface-area MgO sorbents, catalysts or catalyst supports after calcination [37,39]. When combined with brine-derived magnesium, hydrothermal shaping therefore provides an enabling step to transform a low-value commodity hydroxide into application-tailored materials with performances comparable to those prepared from high-purity precursors [27,36,45,46].

Solvothermal synthesis extends the same principles to organic solvents or water–organic mixtures, exploiting different solvent polarity, coordination ability, and dielectric properties to further control Mg(OH)_2_ nucleation and growth [1,47]. For example, a simple solvothermal technique in alcohols produced single-crystalline Mg(OH)_2_ nanotubes and related nanostructures, with the crystallite shape and structure being finely controlled by solvent choice, concentration, and reaction temperature; the lower polarity and distinct solvation environment relative to water were crucial to stabilizing tubular morphologies [48]. Mixed aqueous/organic systems can be viewed as intermediate between hydrothermal and solvothermal conditions and have been exploited to tailor Mg(OH)_2_ texture without fully switching to non-aqueous solvents. As mentioned above, altering the water/ethanol ratio switched the product from nanowires in pure water to micro-flowers in mixed medium, implying that solvent polarity, hydrogen bonding network, and mass transport properties jointly govern the anisotropic growth and self-assembly of brucite crystallites [37]. Organic solvents also enable better dispersion of hydrophobic additives or templates and, in some routes, act as both solvent and weak base or ligand, influencing Mg speciation and growth kinetics [38,48,49]. From the standpoint of brine valorization, solvothermal methods are less straightforward than hydrothermal ones because of the need to remove large amounts of inorganic electrolyte and to adapt to the corrosion and safety requirements of organic solvents at high temperature [45,47]. Nevertheless, they offer niche possibilities for producing highly specialized Mg(OH)_2_ and MgO nanomaterials (e.g., nanotubes, hollow spheres, or core–shell architectures) that could not be easily obtained in purely aqueous systems, potentially using brine-derived Mg intermediates that have already been purified and concentrated [50,51]. Compared with direct ambient temperature precipitation from brines, hydrothermal and solvothermal syntheses decouple chemical purification from structural tailoring and allow much finer control of morphology, crystallinity, and defect structure at the expense of higher energy input and batch-type operations. Direct precipitation in continuous reactors such as MF PFRs or controlled granulometry crystallizers can already achieve high Mg recovery and cationic purity from favorable feeds like saltwork bitterns but often yields broad particle size distributions and partially defective solids; a subsequent hydrothermal step can refine these powders into application-ready materials without significantly altering bulk composition. In contrast, solvothermal methods are best viewed as specialty routes for producing nanostructured Mg(OH)_2_ from already purified magnesium streams, enabling elaborate morphologies but adding solvent handling and recovery complexity. Overall, the combination of brine-based Mg extraction, controlled precipitation, and hydrothermal/solvothermal shaping offers a coherent pathway from waste brines to high-value Mg(OH)_2_ nanomaterials. Future work is likely to focus on integrating these steps in semi-continuous or continuous architectures, optimizing energy efficiency, and coupling hydrothermal aging directly to upstream brine mining processes, while systematic studies on the interplay between brine impurities, precursor structure, and hydrothermal/solvothermal conditions will be essential to translate laboratory syntheses into scalable, industrially robust routes.

### 2.4. Sol–Gel Method

The sol–gel process relies on the controlled transformation of molecular or ionic precursors into an inorganic network through hydrolysis and condensation reactions, forming first a colloidal sol and then a continuous gel. The method could be exploited as a complementary route to recover magnesium hydroxide when a higher degree of structural control and functionalization is required compared to conventional precipitation [52]. In a recovery framework, magnesium-bearing streams (such as brines, industrial effluents or process liquors) can be converted into a colloidal solution of magnesium-containing precursors, which subsequently undergo hydrolysis and condensation reactions to form an interconnected gel network incorporating Mg(OH)_2_ at the nanoscale [53]. This pathway would allow the transformation of dilute or impure magnesium sources into value-added Mg(OH)_2_-based materials, rather than simply generating a bulk precipitate to be separated and disposed [54]. From an application-oriented perspective, using sol–gel processing for Mg(OH)_2_ recovery would provide several potential advantages. First, it enables the synthesis of nanostructured, highly dispersed Mg(OH)_2_ with tunable particle size, porosity and surface area, which can be beneficial for catalytic, adsorptive or flameretardant applications. Second, the gel state offers a versatile platform to incorporate Mg(OH)_2_ into hybrid organic–inorganic matrices, coatings or composite particles directly during the recovery step, simplifying downstream processing. Finally, by tailoring solvent systems, templating agents and drying/calcination protocols, the sol–gel route can convert recovered magnesium into engineered Mg(OH)_2_ or MgO materials with specific textural and interfacial properties, thus upgrading a waste-derived feedstock into a high-performance functional product. Magnesium hydroxide MDH is also increasingly synthesized via sol–gel routes to obtain nano- and microstructured powders, thin films and hybrid coatings with controlled porosity, particle size and surface chemistry for flame retardancy, catalysis and biomedical applications [52]. For magnesium systems, this transformation typically proceeds via hydrolyzed Mg–OH species that condense into Mg–O–Mg linkages, with solvent composition, pH, complexing ligands and temperature governing nucleation, growth and eventual pore structure of the dried gel. Sol–gel synthesis of Mg-containing phases uses a range of precursors, including inorganic salts such as Mg(NO_3_)_2_ and MgCl_2_, magnesium alkoxides, and mixed metal alkoxides when preparing Mg-containing composite oxides or coatings. Complexing agents (e.g., citric acid, polyols, or amino alcohols) and mixed aqueous–organic solvents are employed to stabilize Mg^2+^ in solution, moderate hydrolysis rates and prevent premature precipitation, thereby enabling homogeneous sols and finely dispersed gel networks. In typical Mg(OH)_2_ sol–gel synthesis, an aqueous or mixed-solvent solution of the magnesium precursor is slowly neutralized or basified in the presence of stabilizing ligands, generating nanometric Mg-containing nuclei that remain dispersed as a sol rather than aggregating into bulk precipitate. Upon aging at moderate temperatures (often 60–80 °C) and controlled ionic strength, these primary particles undergo further condensation and assembly, leading to the formation of a percolating gel network whose connectivity and pore size depend on precursor concentration, base/metal ratio and solvent polarity [52,53,54].

The drying of Mg-based gels (via ambient, supercritical or freeze-drying) removes solvent while largely preserving the nanostructured skeleton, producing xerogels or aerogels that can contain amorphous or poorly crystalline Mg(OH)_2_–MgO domains [55]. Subsequent thermal treatments between roughly 200 and 500 °C can tune the balance between Mg(OH)_2_ and MgO, crystallinity, pore structure and specific surface area, with higher temperatures favoring MgO formation and densification but potentially reducing accessible porosity [56]. Dedicated sol–gel routes have produced size- and shape-controlled Mg(OH)_2_ nanostructures at temperatures below 80 °C by carefully selecting oil–water solvent systems and precursor/base ratios, yielding highly crystalline, mesoporous powders [53,54]. These nanostructures can exhibit high specific surface area and tunable particle size distributions (from tens to a few hundred nanometers), which is advantageous for adsorption, catalysis and as reactive precursors to MgO with controlled grain size [57].

The morphology of sol–gel-derived Mg(OH)_2_, whether quasi-spherical nanoparticles, anisotropic nanoplates or mesoporous aggregates, is determined by the interplay of hydrolysis rate, condensation kinetics, ligand binding and drying route [55]. These structural features in turn control surface area, pore size distribution, basicity and dispersion in polymer matrices or biological media, governing performance in flame retardancy, adsorption, catalysis and biomedical delivery. Sol–gel-derived MgO nanoparticles and related Mg(OH)_2_-based materials are being developed as controlled Mg^2+^ carriers for biomedical applications, including osteoarthritis treatment, where release kinetics and biocompatibility depend strongly on particle size and surface chemistry [58]. Although sol–gel techniques are not yet the primary choice for the large-scale valorization of desalination brines, combining brine-derived magnesium salts with sol–gel chemistry could enable high-value nanostructured Mg(OH)_2_ and MgO products (e.g., functional fillers and coatings) as a boutique downstream route. Such integration would require the pre-purification of brine Mg^2+^, control of competing ions and careful solvent and ligand management, but could complement mass-production precipitation with a smaller, high-margin sol–gel line.

### 2.5. Ball Milling Mechanochemical Technique

Ball milling could be employed as a mechanical processing route to recover and upgrade magnesium hydroxide from secondary or sub-standard sources, such as industrial byproducts, undersized fractions from filtration, or agglomerated precipitates [59,60]. In this context, Mg(OH)_2_ recovered via reactive crystallization often exhibits strong aggregation, broad particle size distributions and poor dispersibility, which limit its direct use in high-performance applications [61]. Applying ball milling to these powders would allow the controlled reduction in particle size, the deagglomeration of clusters and the homogenization of the material, transforming a heterogeneous, difficult-to-handle solid into a more uniform and processable product [62,63].

From a functional standpoint, the use of ball milling for Mg(OH)_2_ recovery offers multiple advantages [64]. Mechanical activation can increase the specific surface area and modify surface defects, potentially enhancing the reactivity of Mg(OH)_2_ in applications such as neutralization, adsorption or as a precursor to MgO. Moreover, ball milling provides a straightforward way to produce composite or hybrid powders by co-milling Mg(OH)_2_ with polymers, other inorganic fillers or additives, thereby integrating the recovered material directly into formulations for flame-retardant systems, composites or pellets. By appropriately tuning milling parameters (time, rotational speed, ball-to-powder ratio, use of wet vs. dry milling), the technique can be adapted either to gently refine particle morphology and dispersibility or to strongly activate and functionalize the recovered magnesium hydroxide, adding value to an otherwise low-grade or waste-derived feedstock [65]. High energy ball milling is a mechanochemical technique in which powders are repeatedly impacted by grinding media inside a rotating mill, causing severe plastic deformation, fracture and cold welding that collectively generate nanostructured or amorphous solids. For magnesium hydroxide and related Mg-based systems, ball milling is used to activate otherwise stable phases, refine particle and grain size, introduce defects and, in some cases, drive solid-state reactions such as hydride formation or layered double hydroxide (LDH) synthesis [66,67]. Repeated impacts in a ball mill produce extremely high local stresses and strain rates, which fragment particles, create fresh surfaces and accumulate dislocations and other crystallographic defects. As milling proceeds, the microstructure evolves through stages of particle refinement, grain subdivision and partial amorphization, increasing the free energy of the solid and reducing kinetic barriers for diffusion-controlled processes and phase transformations. In Mg(OH)_2_-based systems, pre-milling has been shown to transform a well crystallized brucite structure into a finely divided, partially amorphous state that is highly reactive toward other hydroxides and oxides in the solid state. This activated Mg(OH)_2_ can, for example, react completely with Al(OH)_3_ under continued milling to form Mg–Al layered double hydroxides, a transformation that is not feasible at comparable rates without the mechanochemical input provided by the mill [68]. Detailed studies on Mg powders provide insight into the generic evolution under ball milling: an initial regime where particle size drops rapidly and morphology changes from flakes to more equiaxed fragments, followed by a regime of nanograin formation and defect accumulation. At longer milling times, a steady-state nanocrystalline structure is often reached, where further input mainly reorganizes defects and sub-grains rather than significantly reducing grain size, with the final microstructure governed by the balance between deformation and dynamic recovery. For composite systems containing MgO, Mg(OH)_2_ and associated carbonate species (e.g., hydro magnesite), ball milling can generate intimately mixed, nanoscale dispersions, core–shell structures or defect-rich aggregates with tailored thermal and gas–solid reactivity [66]. Mechanochemically processed Mg-based powders are central to hydrogen storage research, because grain refinement and defect engineering substantially accelerate hydrogen absorption and desorption, although further work is needed to reduce operating temperatures for integration with fuel cell systems [69].

In polymer composites and flame-retardant systems, pre-milled Mg(OH)_2_ improves dispersion and can be co-milled with polymers or co-additives, enabling in situ formation of hybrid structures or fine blends that enhance flame retardancy and mechanical performance [64,65,70]. In a process chain where Mg(OH)_2_ is first recovered from brines by precipitation or hydrothermal treatment, ball milling is best regarded as a downstream, high-energy post-processing step applied to selected fractions destined for high-value applications such as hydrogen storage, catalysis or specialty fillers [62,63]. Process integration requires quantitative evaluation of mill energy consumption, throughput and long-term stability of the nanostructured state, but the current literature on mechanochemically engineered Mg-based materials indicates that ball milling is a powerful tool for tailoring structure and performance beyond what is achievable by purely thermal or solution-based methods [69]. Ball milling is also highly relevant when magnesium hydroxide is obtained from natural brucite derived from quarrying activities. In this case, the starting material is typically produced by mining, crushing and grinding brucite rock, followed by beneficiation and, in some processes, controlled hydration to yield Mg(OH)_2_ powders [71]. These quarry-derived products often show relatively large particle sizes, irregular shapes and a tendency to form aggregates that can impact dispersion in polymer matrices and the overall performance as a flame-retardant filler. Applying ball milling to such Mg(OH)_2_ enables further comminution and refinement of the particle size, breaking down aggregates and smoothing extreme morphological irregularities. As a result, the recovered filler becomes more compatible with common compounding operations, improving its distribution within the polymer and reducing defects or weak points in the final composite [64]. In terms of effectiveness, the milling of quarry-derived brucite-based Mg(OH)_2_ can substantially influence both processing behavior and end-use properties. On the processing side, a more controlled and narrower particle size distribution improves flowability, dosing accuracy and mixing efficiency in extruders and internal mixers, while also helping to reduce sedimentation issues in liquid formulations. At the application level, appropriate milling conditions can enhance the balance between specific surface area and particle size: smaller, better-dispersed particles tend to provide more uniform flame-retardant action, improved mechanical properties (e.g., tensile strength, elongation at break, impact resistance) and better surface finish in molded articles. However, the milling intensity must be carefully optimized; excessive comminution can produce overly fine particles with high surface energy, leading to re-agglomeration, increased viscosity in polymer melts and potential negative effects on processing and mechanical performance [72]. Therefore, for brucite-derived Mg(OH)_2_, an effective milling strategy is not simply “as fine as possible,” but rather the definition of a target granulometry and morphology that match the requirements of specific formulations, maximizing the added value of the natural resource while keeping energy consumption and processing challenges under control [64,72].

### 2.6. Innovative Methods (Microwaves, Ultrasounds and Microemulsions)

Innovative processing methods such as microwave-irradiation-, high-intensity-ultrasound- and microemulsion-assisted synthesis are increasingly investigated and described in Table 1 as complementary routes to the conventional precipitation and hydrothermal production of magnesium hydroxide, with the aim of intensifying reaction kinetics and exerting finer control over particle size, morphology and dispersion. Microwave-assisted protocols exploit the volumetric, selective heating of Mg^2+^/OH^−^ solutions and sol–gel precursors, enabling very rapid nucleation and growth of Mg(OH)_2_, and allowing size- and shape-controlled nanoparticles, thin films and composite particles to be obtained in minutes rather than hours [39,53,55,73]. High-intensity ultrasound introduces acoustic cavitation into reacting Mg^2+^/OH^−^ systems, producing transient hot spots, shock waves and microjets that intensify local mixing and mass transfer. In seawater and Mg-rich process waters, ultrasound-assisted precipitation has been associated with enhanced nucleation rates, improved dispersion of nuclei and reduced tendency toward uncontrolled agglomeration, ultimately leading to smaller mean particle sizes and narrower size distributions of Mg(OH)_2_. Pilot scale studies on magnesium recovery from brines, although not always using ultrasound explicitly, show that intensified mixing strategies can significantly influence Mg(OH)_2_ particle characteristics and reagent consumption, providing a conceptual framework for deploying ultrasound as a process intensification tool in brine treatment schemes. These findings suggest that coupling acoustic fields with chemical precipitation could be particularly valuable in applications where fine control of Mg(OH)_2_ particle size and morphology is required directly at the recovery step [9,74]. Microemulsion-based and related soft chemistry routes exploit nanometer scale aqueous droplets dispersed in oil–surfactant media as confined reactors for Mg^2+^/OH^−^ reactions, offering precise control over nucleation volume and growth conditions. In such systems, the composition of the microemulsion (water to oil ratio, surfactant type and concentration) governs the size and connectivity of the aqueous domains, enabling the synthesis of porous MgO from Mg(OH)_2_ precursors with tunable pore structure and surface area through mild post-treatments. Beyond simple oxides, sol–gel and microemulsion strategies have been used to produce polymer/Mg(OH)_2_ core–shell particles and composite powders, where Mg(OH)_2_ (or MgO) is intimately integrated with organic matrices such as polystyrene, resulting in highly dispersible fillers with well defined shell thickness and controlled inorganic core size. Hybrid sol–gel coatings incorporating Mg species have also been engineered on metallic substrates, including magnesium alloys, to improve corrosion resistance and interfacial performance by designing multilayer, organic–inorganic architectures with tailored composition and crosslinking. Taken together, these microemulsion and soft chemistry approaches demonstrate how interfacial and confinement effects can be harnessed to design Mg(OH)_2_-based nanostructures and composites with sophisticated morphologies and functionalities that complement bulk precipitation products [52,55]. In Table 2, a summary of the production methods is reported.

## 3. Surface Modifications and Functionalization

Surface modification of magnesium hydroxide is one of the most recurrent themes in the Mg(OH)_2_ literature, particularly in the context of polymer composites and flame-retardant formulations [75]. The structure of Mg (OH)_2_ exposes a high density of surface hydroxyl groups OH, which provide anchoring sites for covalent or physical interactions, making this hydroxide an intrinsically highly functionalizable inorganic filler. Tailoring the surface chemistry of Mg(OH)_2_ is crucial to improve interfacial adhesion with polymer matrices, control surface polarity and hydrophobicity, enhance dispersibility under melt-processing conditions, introduce specific functional groups and suppress particle aggregation both during synthesis and compounding [76]. Silane coupling agents such as vinyltriethoxysilane (VTES), 3 methacryloxypropyltrimethoxysilane (A 174) and n octyltriethoxysilane are commonly grafted or adsorbed onto Mg(OH)_2_, converting the originally hydrophilic surface into a more organophilic one and thereby increasing compatibility with nonpolar matrices such as polypropylene (PP), polyethylene (PE) and ethylene–vinyl acetate (EVA). Lan et al. showed that silane modification of Mg(OH)_2_ by a dry process effectively improves its dispersion and interfacial bonding in polymer composites [77], whereas Wang et al. reported that A 174 treatment leads to enhanced mechanical properties and processability at high filler loadings [78]. Industrial flame-retardant grades often combine silane treatments with fatty acids or metal soaps (e.g., stearic acid), which further decrease surface energy, improve powder flow and facilitate homogeneous filler distribution without severely compromising the flame-retardant performance [79].

**Figure 9:**
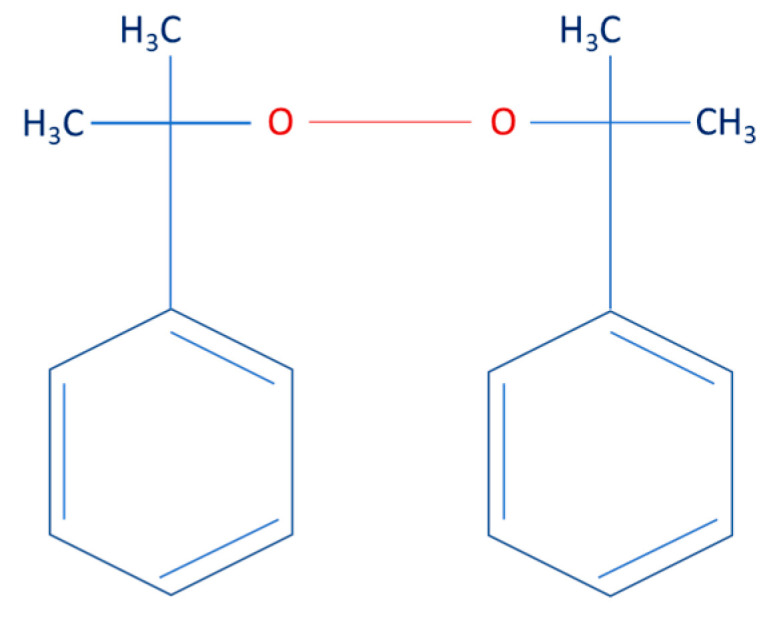
Chemical structure of the MA-grafting mechanism precursor.

In parallel with direct mineral modification, it is important to mention also the surface engineering of the polymeric phase through maleic anhydride that provides a complementary and, in many commercial systems, synergistic route to optimize the Mg(OH)_2_ polymer interface. In polyolefin-based composites, maleic-anhydride-grafted polypropylene PP g MAH or maleic-anhydride-grafted linear low-density polyethylene LLDPE g MAH is routinely used as a compatibilizer; the polar anhydride groups can interact with hydroxylated Mg(OH)_2_ surfaces, while the polyolefin backbone remains miscible or co-crystallizes with the matrix, thus reducing interfacial tension and promoting stress transfer. Zhu et al. and Liu et al. demonstrated that combining Mg(OH)_2_ with PP g MAH in PP composites leads to improved dispersion, higher tensile strength and better flame-retardant performance compared with non-compatibilized systems, confirming that polymer-grafted maleic anhydride behaves as an effective interfacial modifier around the inorganic filler as [80]. More recent studies on LLDPE/Mg(OH)_2_ systems proves that maleic-anhydride-grafted compatibilizers enhance toughness and elongation at break while preserving the high limiting oxygen index required for halogen-free flame retardancy [81]. These compounds are typically prepared by melt free-radical grafting in the presence of an organic peroxide initiator, most commonly dicumyl peroxide (DCP), shown in Figure 9, using batch mixers or, more relevantly for applications, twin-screw reactive extrusion. Under melt conditions, the peroxide decomposes to generate macroradicals on the polyolefin backbone, which then add maleic anhydride (MA) units; in parallel, side reactions such as β scission, chain branching/crosslinking, and MA homopolymerization occur, so the process is optimized to maximize grafting while minimizing degradation [82].

**Figure 10:**
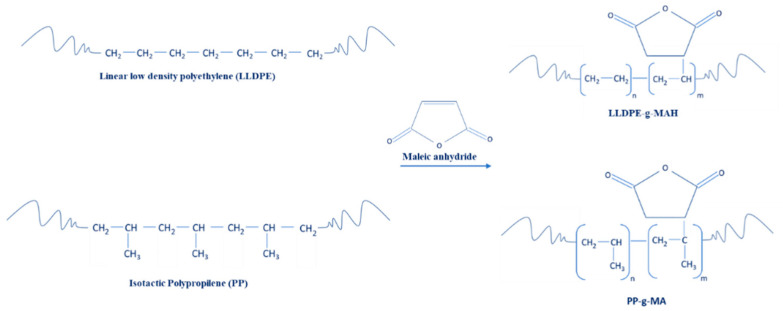
MA-grafting mechanism.

For polypropylene, isotactic PP (granular or powder) is fed with MA and DCP into an internal mixer or into a corotating twin-screw extruder, typically at barrel temperatures around 150 °C, residence times of the order of min in batch or depending on the screw rotational speed and length in extrusion, and also the screw configurations are designed to ensure intensive mixing but limited residence time. Systematic studies show that the degree of grafting increases with MA and peroxide content up to an optimum, then decreases due to competing degradation and MA homopolymerization; for example, an “optimal” formulation around 5 phr MA and 0.3 phr DCP gives high grafting efficiency with acceptable molecular weight reduction, while increasing either component further leads to more pronounced chain scission and lower melt strength. In reactive extrusion, the reaction mechanism of which is shown in Figure 10, of powder and granular PP, the grafted MA content can be tuned between about 0.02 and 0.5 mol% (corresponding roughly to 0.1–1.5 wt% MA) by adjusting MA and DCP feed; powder PP generally exhibits higher grafting efficiency than granular PP due to better initial mixing and reduced diffusional limitations. The grafted MA content is routinely quantified by FTIR spectroscopy (carbonyl bands at ≈1780–1850 cm^−1^) and acid–base titration after hydrolysis of anhydride rings, while GPC and rheometry are used to monitor molecular weight loss and changes in melt flow index [82,83].

**Figure 11:**
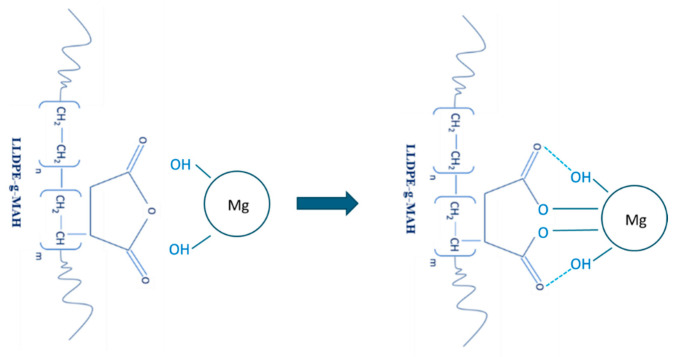
MA-bonding close-up.

In the case of LLDPE, the same radical mechanism is employed as described in Figure 11, but the lower crystallinity and different comonomer distribution can alter radical stability and diffusion, so processing conditions are often tuned specifically for this matrix. A widely cited route consists of a two-step protocol: first, LLDPE is pre-irradiated (e.g., UV or γ) to form hydroperoxide groups along the chains, and second, the pre-activated LLDPE is processed with MA in a reactive extrusion step where these hydroperoxides decompose thermally, yielding radicals that graft MA without the need for additional peroxides; reactive extrusion is typically conducted at 170–190 °C with MA contents of a few wt% and residence times in the sub-minute range [84]. FTIR, DSC and solid-state NMR confirm the presence and distribution of grafted MA units on LLDPE, and titration or FTIR calibration curves are used to determine graft levels, which for compatibilizer applications are again commonly in the 0.5–1.5 wt% range. Beyond conventional peroxide-initiated grafting, a more recent “flash reactive extrusion” approach has shown that PE (and by extension LLDPE grades) can be maleated even in the absence of added peroxides by processing at very high melt temperatures (up to ≈420 °C), where thermal degradation generates macroradicals that react with MA, achieving grafting degrees around 1.4 wt% at 3 wt% initial MA with grafting yields of ≈50% [84,85].

**Figure 12:**
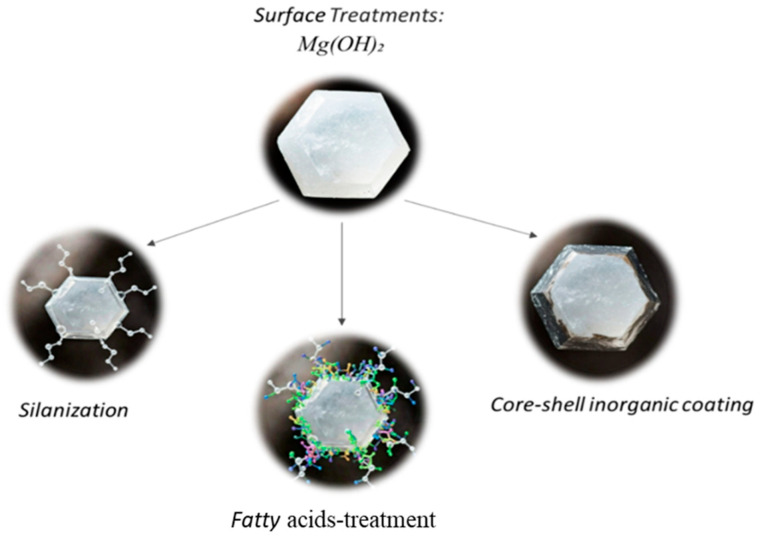
Possible surface modification of magnesium hydroxide (MDH).

State-of-the-art formulations therefore tend to combine both levels of modification: a primary surface treatment on Mg(OH)_2_ (typically silane- and/or stearate-based) and a secondary compatibilization of the polymer phase via maleic-anhydride-grafted polyolefins, resulting in a finely tuned interphase region [86]. The patent- and application-oriented literature indicates that such hybrid strategies schematized in Figure 12 are already implemented at industrial scale in wire-and-cable, automotive and building applications, where the balance between processability, mechanical integrity and flame-retardant efficiency is tightly constrained by standards and regulations [77,81].

### 3.1. Silanization Treatment

**Figure 13:**
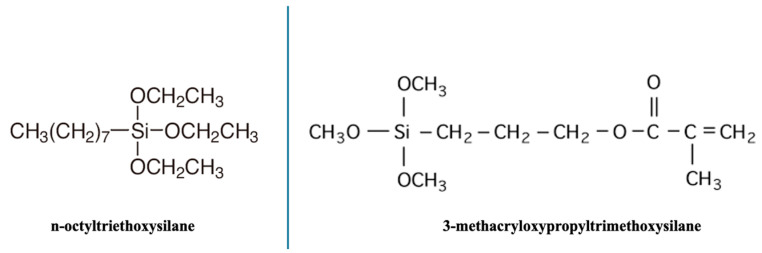
Chemical structure of common organosilane used in the silanization treatment.

Silanization of magnesium hydroxide is widely recognized as the most common strategy to tailor the interfacial chemistry of Mg(OH)_2_ in polymer composites, because the high density of surface hydroxyl groups enables the formation of robust chemical or hydrogen bonds with organosilane coupling agents. In typical formulations, trialkoxysilanes such as vinyltriethoxysilane (VTES), 3 methacryloxypropyltrimethoxysilane (A 174) or n octyltriethoxysilane (chemical sructures shown in Figure 13) are selected to introduce specific organic functionalities (vinyl, methacrylate, alkyl) that are compatible with the target polymer matrix, while the alkoxy groups undergo hydrolysis–condensation reactions with Mg–OH sites on the filler surface [75,77,78,79].

**Figure 14:**
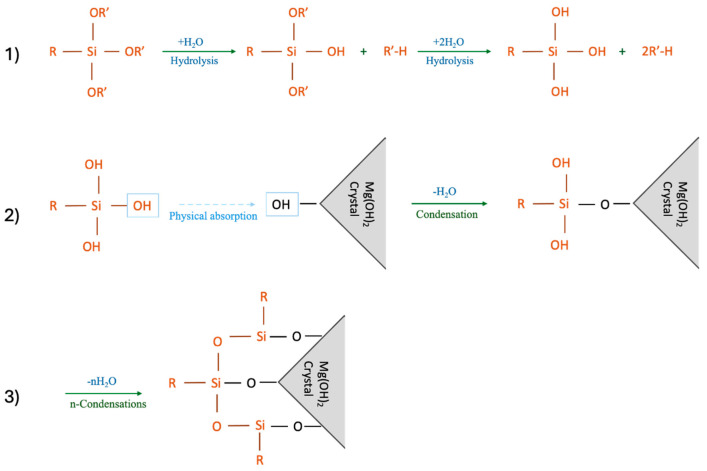
Silanization treatment 3-step mechanism.

The silanization of magnesium hydroxide proceeds through a tightly coupled sequence of hydrolysis shown in Figure 14, adsorption and condensation steps, whose kinetics and balance determine the structure and effectiveness of the interphase formed on the filler surface [87]. In the hydrolysis stage, the alkoxy groups of trialkoxysilanes such as vinyltriethoxysilane (VTES) or 3 methacryloxypropyltrimethoxysilane (A 174) are converted into silanols in the presence of water; this step must be carefully tuned in terms of pH, water availability and silane concentration to generate a sufficient population of reactive silanol species while suppressing premature self-condensation in the bulk phase. In more conventional aqueous or semi-aqueous treatments, silanes are often pre-hydrolyzed under mildly acidic to nearly neutral conditions for a defined time to obtain a stable silanol solution; this strategy, adopted in several Mg(OH)_2_ nanocomposite formulations, reduces bulk gelation and improves the transport of monomeric or low oligomeric silanols to the particle surface [88,89].

Once hydrolyzed, silane species adsorb onto the hydroxylated Mg(OH)_2_ surface, which is rich in terminal OH groups and defect sites that act as anchoring points [77]. The initial adsorption stage is dominated by hydrogen bonding and polar interactions between silanols (depending on the silane, amino or methacrylate functional groups) and Mg(OH)_2_ surface hydroxyls, giving rise to a physically adsorbed layer. The final condensation step involves two cooperative reactions: the formation of Mg–O–Si bonds through condensation between surface hydroxyls and silanols, and the lateral condensation of neighboring silanols into a crosslinked siloxane (Si–O–Si) network. In the VTES/Mg(OH)_2_ system, FTIR and XPS data reveal characteristic Si–O–Mg signals together with Si–O–Si bands, while thermogravimetric analysis shows a small but definite chemically bound organic fraction, supporting a mechanism in which hydrolyzed VTES reacts directly with Mg(OH)_2_–OH groups during the heat treatment to form a thin organosilane shell. Lan et al. emphasized that this shell is sufficiently crosslinked to resist desorption and mechanical detachment, yet it is thin enough to retain the intrinsic crystal structure and thermal decomposition behavior of Mg(OH)_2_, which is essential for preserving its endothermic dehydration and MgO barrier formation in flame retardant applications [77]. In the A 174 system, Wang et al. systematically varied silane loading, temperature and shear and identified optimal conditions (about 1.5 wt% A 174, 145 °C, 10 min, 3000 rpm) that maximize the degree of condensation and yield a dense methacrylate functional siloxane network on the Mg(OH)_2_ surface; under these conditions, water contact angle and dispersion performance are greatly enhanced, highlighting the direct link between interfacial condensation kinetics and macroscopic surface properties [78]. Cabrera Álvarez et al. showed that triethoxy vinyl silane (TVS)-treated Mg(OH)_2_ in HDPE nanocomposites still requires high filler contents to achieve self-extinguishing behavior, but the detrimental effect of Mg(OH)_2_ on tensile properties is markedly reduced; while unmodified Mg(OH)_2_ leads to hard, brittle materials with strongly decreased flexibility, silane-modified Mg(OH)_2_ yields composites with higher tensile strength and elongation at break at comparable loadings. In the same work, all HDPE/Mg(OH)_2_ materials (modified and unmodified) passed UL 94 HB, but flame permanence time was slightly longer when TVS was used, indicating that the main benefit of silanization in this system is mechanical reinforcement and improved dispersion rather than a direct increase in intrinsic flame retardant efficiency [89].

Future developments in silane-modified magnesium hydroxide composites are expected to focus on more integrated control of particle morphology, surface chemistry and multi-scale structure to decouple, as far as possible, the high mineral loading and loss of mechanical performances. Recent work on modifier-directed precipitation and one pot synthesis of hexagonal or nanoscale Mg(OH)_2_ indicates that combining in situ silanization with morphology control can yield fillers that are intrinsically more dispersible and require lower loadings to achieve equivalent flame retardant performance, which is particularly attractive for next-generation lightweight and thin wall components [90]. Future studies may move beyond single silanes toward hybrid coupling strategies that combine silanes with phosphorus, nitrogen or ceramic-forming agents to tailor both the condensed-phase barrier and the gaseous-phase flame retardant mechanisms, while maintaining good rheological behavior in processing [91,92,93,94].

### 3.2. Fatty Acid to Metal Soap Organic Modifications on Mg(OH)_2_

**Figure 15:**
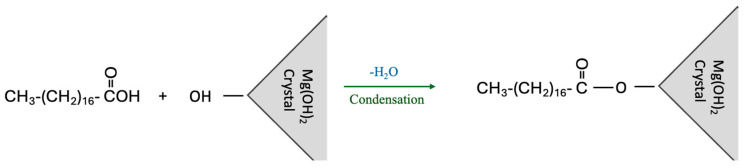
Fatty acid bonding.

Industrial flame-retardant magnesium hydroxide grades commonly use hybrid coating in which a chemically anchored silane layer is combined with low-surface-energy organic treatments based on fatty acids or metal soaps. Fatty acids are among the most versatile organic treatments used to tailor the surface of Mg(OH)_2_, and stearic acid remains the archetype. Once adsorbed or converted into stearate at the hydroxylated Mg(OH)_2_ surface, the mechanism of which is described in Figure 15, it organizes into a hydrophobic hydrocarbon layer that sharply lowers surface energy and screens polar surface sites, thereby weakening particle–particle attractions. Stearic acid forms coordinated carboxylate species and relatively ordered alkyl chains, building a compact organic shell that improves powder flowability and reduces slurry and compound viscosity [95,96].

The industrial and patent literature makes it clear that stearic acid is only one member of a broader “toolbox” of higher fatty acids used on Mg(OH)_2_, including oleic acid selected according to the target matrix and processing window [97]. By tuning chain length and degree of unsaturation, formulators can adjust melting behavior, lubricity and compatibility with specific polymers; more crystalline saturated acids favor strong lubrication and thermal stability, whereas unsaturated acids such as oleic acid can confer a slightly softer, more flexible interphase that better follows large deformations. In polyolefin and rubber compounds, these fatty-acid-derived layers promote finer dispersion and suppress hard agglomerates, which directly translates into less brittle behavior and allows higher Mg(OH)_2_ loadings without the dramatic loss of toughness associated with unmodified mineral fillers [98]. In Table 3 the optimal ultrasonic treatment conditions for surface treatment processes for magnesium-based filler with stearic and oleic acids is summarized. For stearic acid, a dosage of 1.25 wt% at 70 °C for 10 min yielded a water contact angle of 109.5°, which is the key parameter used to determine the performance of the process. In contrast, oleic acid required a higher dosage of 7 wt% and a longer treatment time of 3 h at 60 °C to achieve a slightly lower contact angle of 106°.

**Figure 16:**
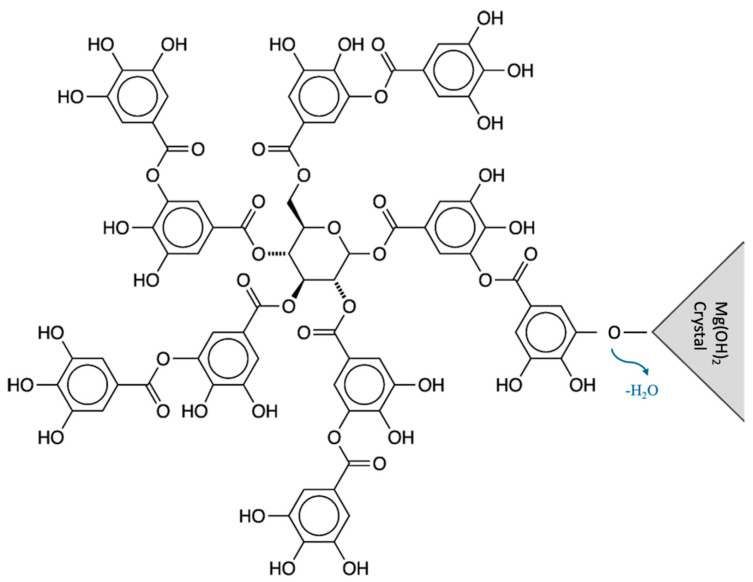
TA structure bonded onto MDH crystal.

Alongside classical long chain fatty acids, polyphenolic species such as tannic acid (Figure 16) emerge as more sophisticated organic modifiers for Mg-based substrates. Thanks to its multiple phenolic and carboxylic groups, tannic acid can chelate the magnesium and interact strongly with hydroxide surfaces, forming robust organic interphases that not only alter wetting and interfacial energy, but also introduce antioxidant, antimicrobial or char-promoting functions [99]. Recent work on magnesium and layered hydroxide hybrids shows that tannic-acid-based coatings markedly improve corrosion resistance and interfacial stability in aqueous environments, and tannic acid intumescent systems demonstrate efficient char formation and reduced flammability, suggesting that TA-modified Mg(OH)_2_ could be engineered to couple halogen-free flame retardancy with additional protective or bioactive roles [100].

### 3.3. Inorganic Co-Fillers for a Modified Ceramic Layer

Unlike purely organic treatments, inorganic shells’ core–shell structures based on phosphates, carbonates or oxides can actively participate in condensed-phase flame retardant mechanisms, promoting cohesive, refractory residues and improving the integrity of the protective layer under fire exposure. In this sense, the design of Mg(OH)_2_-based fillers increasingly involves a dual strategy, where inorganic coatings are combined with organic modifiers to couple processability with robust high temperature performance [101]. Phosphate-containing systems have attracted considerable attention because they can transform into stable, ceramic-like magnesium phosphate phases with excellent fire resistance and high-temperature integrity. In Mg(OH)_2_-containing flame-retardant systems, phosphates can act as acid source or glass-forming components that, upon heating, react with MgO (generated by Mg(OH)_2_ decomposition) to form magnesium phosphate networks, thereby densifying the char and improving its mechanical integrity and adhesion to the underlying substrate or polymer. Synergistic formulations combining Mg(OH)_2_ with phosphorus-based flame retardants, such as red phosphorus, have demonstrated enhanced condensed-phase action in polypropylene, where the inorganic–phosphate residue forms a more continuous barrier that slows heat and mass transfer and strengthens the char structure compared with systems containing Mg(OH)_2_ alone by studying the degradation kinetics with the Kissinger method. This behavior suggests that a designed phosphate coating on Mg(OH)_2_ could pre-organize the chemistry and morphology of the eventual magnesium phosphate layer, offering a route to finely tuned barrier properties without excessively increasing the total phosphorus content of the composite [102].

**Figure 17:**
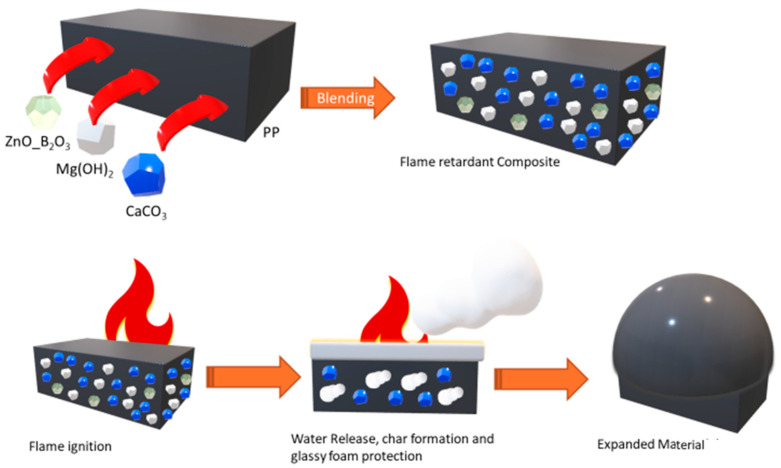
Inorganic co-filler flame retardancy ability in PP matrix.

Carbonates involving magnesium hydroxide have also been explored mainly in the form of inorganic core–shell fillers and co-filler FR formulations. A representative example is the preparation of a calcium carbonate core magnesium hydroxide shell composite filler specifically designed as an inorganic composite flame retardant for polymers. In this process, precipitated CaCO_3_ is dispersed in water and reacted with a soluble magnesium salt (e.g., MgSO_4_) in the presence of Ca(OH)_2_, so that Mg(OH)_2_ nucleates and grows on the CaCO_3_ surface, forming a core–shell structure; the product is then filtered, dried and further surface modified using a silane and stearic acid to improve compatibility with polyolefin matrices. The resulting filler combines the low cost and high stiffness of CaCO_3_ with the endothermic, smoke-suppressing flame retardant function of Mg(OH)_2_, and is explicitly claimed to provide “filling enhancement, flame retardancy and smoke elimination” in polyolefins and PVC while reducing the dosage of expensive Mg(OH)_2_ compared with formulations based on pure MH. Co-filler systems combining CaCO_3_ and Mg(OH)_2_ are better documented quantitatively, even if they are not always described explicitly as coated structures. A recent study on polypropylene composites containing calcium carbonate and magnesium hydroxide-based flame retardants showed that adding Mg(OH)_2_ and zinc borate to a PP/CaCO_3_ matrix can significantly boost flame retardancy without catastrophic loss of impact properties. In that work, PP/CaCO_3_ samples with a fixed CaCO_3_ loading were modified with varying amounts of Mg(OH)_2_ and Zn borate; formulations with about 10 wt% total flame retardant (Mg(OH)_2_ + Zn borate) exhibited increased limiting oxygen index (LOI), reduced peak heat release rate and lower total smoke production compared with PP/CaCO_3_ without Mg(OH)_2_, while the notched impact strength remained at a level acceptable for engineering applications [103]. This performance described in Figure 17 can be attributed to a synergistic mechanism in which Mg(OH)_2_ decomposes endothermically, releasing water and forming MgO, which cools the material and dilutes flammable gases, whereas the other flame retardant phases promote the formation of a “foamy glassy layer” on the surface of the composite that acts as a thermal and mass transfer barrier during burning. Although CaCO_3_ is not formally a coating on Mg(OH)_2_ here, the intimate contact between CaCO_3_ and MH within the same microstructure and the shared role of CaCO_3_ as a structural, cost-reducing filler make this system a practical example of Mg(OH)_2_–carbonate synergy relevant for real world formulations.

Oxide-based hybrid systems involving magnesium hydroxide are conceptually attractive. The structure can be nominated as Mg(OH)_2_/MO_x_ hybrids, where the second oxide is a distinct phase with its own function. A notable example, cited in the context of core–shell MH systems, is MH@MoO_3_: Dang et al. prepared magnesium hydroxide and molybdenum trioxide flame retardants (MH@MO) and incorporated them into flexible PVC, showing that MH@MO had better synergistic flame-retardant efficiency than either MH or MoO_3_ alone. In this hybrid, the MoO_3_ shell participates in a classical oxide-based vapor-phase/condensed-phase synergy: MoO_3_ can form volatile MoO_2_(OH)_2_ species and promote char formation, while Mg(OH)_2_ provides endothermic cooling and in situ MgO formation; the combination produces a more continuous, thermally stable barrier layer and a stronger reduction in heat and smoke release than either component individually. Quantitatively, although the detailed cone calorimeter data for MH@MO/PVC are not listed alongside those of MH@DOPO@MF, the study reports that MH@MO reduces pHRR and THR more efficiently than unmodified MH at equivalent loadings, illustrating the benefit of adding an oxide shell (MoO_3_) around Mg(OH)_2_ in terms of fire performance [104].

## 4. Industrial Applications of Flame Retardants and Smoke Suppressors: Bulk Application

**Figure 18:**
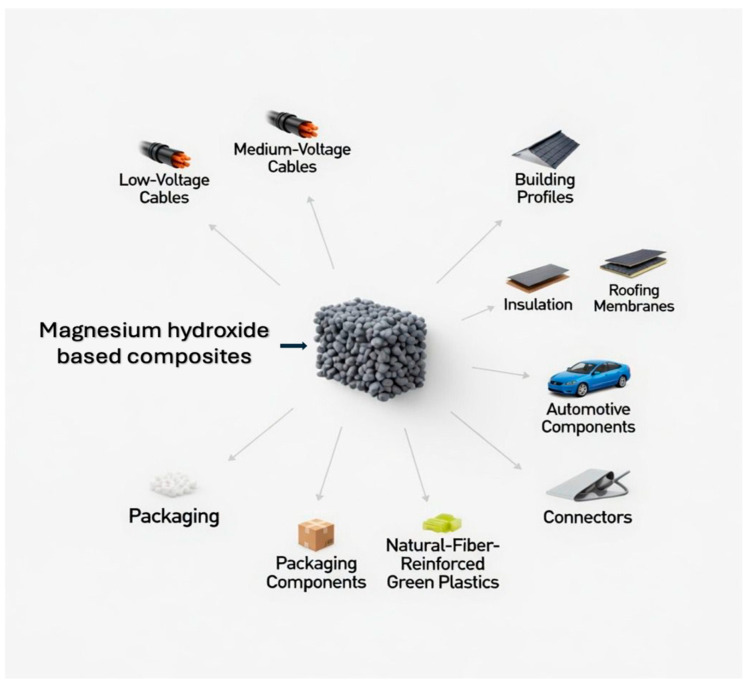
Possible applications for MDH in the formulation of FR composites.

Magnesium hydroxide has evolved to a multifunctional additive for a wide range of industrial polymer composites; its applications are known to be at a very high range as depicted in Figure 18. In polyolefin matrices such as LDPE, LLDPE and PP, Mg(OH)_2_ is a reference halogen-free flame retardant in low-voltage and medium-voltage cable insulation and sheathing, conduits, roofing membranes, geomembranes, appliance housings and building profiles, where its endothermic decomposition, water release and in situ MgO formation provide cooling, dilution of combustible gases and a refractory barrier layer that together deliver self-extinguishing behavior with low smoke and negligible halogen acid emissions [105,106,107,108,109,110,111,112,113,114,115,116,117,118,119,120,121,122,123,124,125,126,127,128,129,130,131,132,133,134,135,136,137,138,139,140,141,142,143,144,145,146,147,148,149,150,151,152,153,154,155,156,157,158,159,160,161,162,163,164,165,166,167,168].

In cable compounds based on PE or PP (often EVA- or EBA-modified), magnesium hydroxide is typically used at high loadings and combined with compatibilizers or surface-treated grades to balance fire performance (LOI, UL-94, IEC cable tests) with the tensile strength, elongation at break and extrusion stability demanded by large-scale wire-and-cable production. Beyond flame retardancy, fine or tailored-morphology Mg(OH)_2_ can act as a structural filler to adjust stiffness, modulus and heat-deflection temperature, and its mild basicity helps neutralize acidic degradation products and catalyst residues, improving long-term insulation performance and weathering resistance in outdoor and electrical polyolefin components [105], and in engineering plastics and industrial thermoplastics such as PA, PBT, PET and PC/ABS blends used in automotive components, E&E housings and connectors. In these systems, Mg(OH)_2_ is often part of hybrid filler packages together with glass fibers, talc or other minerals, where its particle size and surface modification are tuned to provide reinforcement and control shrinkage while contributing to smoke suppression and reduced corrosive gas formation during fire. The ability to apply inorganic (silica, phosphates, oxides) or organic (silanes, fatty acids, metal soaps) coatings on Mg(OH)_2_ has enabled its integration into more polar engineering matrices, allowing formulators to design single fillers that simultaneously improve mechanical performance, processing rheology and fire behavior [77,106]. In elastomeric composites, magnesium hydroxide is a cornerstone of halogen-free flame-retardant (HFFR) technology for flexible materials. EVA and EBA copolymers heavily filled with Mg(OH)_2_ dominate HFFR cable sheathing and insulation formulations in power, telecommunication and data cables, where they must combine high LOI and low smoke with flexibility, fold and bend endurance, and stable extrusion on high-speed lines. In EPDM, EVM, TPV and silicone-based compounds, Mg(OH)_2_ is used for low-smoke, halogen-free rubber jackets, hoses, seals and profiles for automotive, construction and industrial plants; in these matrices, well dispersed, often surface-modified Mg(OH)_2_ can reinforce the elastomer, moderate heat build-up under dynamic loading and provide a coherent inorganic skeleton after burning that supports the remaining char and limits dripping. Its alkaline nature can also help buffer acidic byproducts formed during crosslinking or aging, supporting retention of mechanical properties in long-life sealing and vibration-damping applications [105]. More recently, magnesium hydroxide has gained prominence in biopolymer-based composites, where the industry is seeking high-purity, morphology-controlled, halogen-free, low-smoke solutions that align with sustainability targets and specific applications [107]. In PLA and other bio-polyesters, Mg(OH)_2_ is combined with phosphorus- and nitrogen-containing bio-based flame retardants or intumescent systems to produce materials for packaging and interior components that exhibit improved fire performance, reduced smoke density and better thermal stability during processing [108,109]. In starch-based blends, cellulose-containing biocomposites and natural-fiber-reinforced “green” plastics, Mg(OH)_2_ fulfills multiple functions: it contributes to flame retardancy, buffers acidity that can catalyze hydrolysis of biopolymers, and, in some cases, helps limit odor and VOC emissions, which could be attractive for packaging, furniture and interior building products [110].

**Figure 19:**
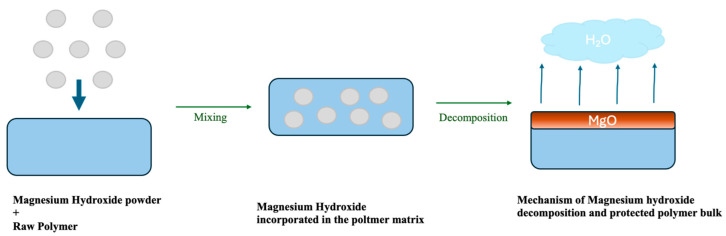
MDH–polymer composite flame retardant properties and mechanism.

The action of magnesium hydroxide can be comprehended by observing the process as the material is heated from ambient conditions up to, and through, ignition, as illustrated in Figure 19. The central chemical event is the endothermic decomposition of Mg(OH)_2_:

$$Mg{\left(OH\right)}_{2\;}\underset{T}{\to }\;MgO+H_{2}O\;$$

This reaction typically begins around 330–340 °C and extends up to roughly 450–500 °C, absorbing a substantial amount of heat from the flame front and from the degrading polymer [105,111,112,164].

**Figure 20:**
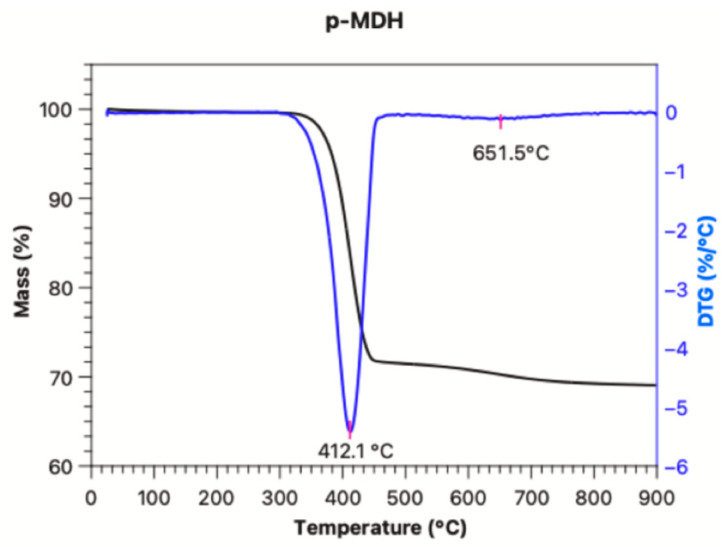
TGA/DTG curve of MDH [61].

The associated heat of dehydration effectively cools the condensed phase, slows the temperature rise in the underlying polymer and delays the onset and rate of pyrolysis, thereby reducing the generation rate of combustible volatiles. The water released appears mainly as vapor and acts as a diluent in the flame zone, mixing with combustion gases and air and lowering the effective concentrations of both fuel and oxygen, which further suppresses combustion and typically leads to reduced burning rate, lower peak heat release rate and increased limiting oxygen index in Mg(OH)_2_-filled composites [113]. In parallel with this gas-phase dilution and cooling, Mg(OH)_2_ decomposes; it leaves behind MgO, which remains in situ at the surface and within the bulk of the polymer. Under fire exposure, MgO particles are often already well dispersed as appropriate surface modification tends to coalesce, partly sinter or bind to the carbonaceous matrix, giving rise to a continuous or semi-continuous inorganic layer that functions as a condensed-phase barrier. This MgO-rich layer reduces thermal conductivity toward the underlying material, slows down the diffusion of oxygen into and volatile degradation products out of the polymer, and helps stabilize any char that forms, improving its cohesion and adherence to the substrate [114]. As a result, dripping, cracking and spalling of the residue are reduced, and the structural integrity of the protective layer is enhanced. In hybrid systems where Mg(OH)_2_ is combined with phosphates, silica or other oxides, MgO can further react to form magnesium phosphates or silico-aluminate-type phases, generating glassy or ceramic-like residues with even better mechanical robustness and barrier efficiency [115]. As already discussed, an important advantage of magnesium hydroxide over halogenated flame retardants and some aromatic phosphorus systems is its benign contribution to the fire effluent. Mg(OH)_2_ does not generate corrosive hydrogen halides or highly toxic aromatic species on decomposition; instead, water vapor and MgO are the primary products. Experimental and application studies on Mg(OH)_2_-filled polymers consistently report lower total smoke production and smoke production rates compared with corresponding halogenated systems, an effect attributed to both the diluting action of water and the barrier action of the MgO/char layer, which inhibit the formation and release of soot-forming fragments [116]. In addition to its intrinsic flame-retardant mechanism, magnesium hydroxide introduces a series of matrix-independent property alterations that are of considerable relevance to composite design. Firstly, the high specific heat and the endothermic dehydration step result in a general stabilizing effect under heat. Thermogravimetric analyses of Mg(OH)_2_-filled polymers frequently demonstrate an elevated onset temperature of degradation and a slower mass loss profile in the TGA and DTG profiles shown in Figure 20. This is indicative of the additional thermal capacity and energy sink associated with water release [61].

**Figure 21:**
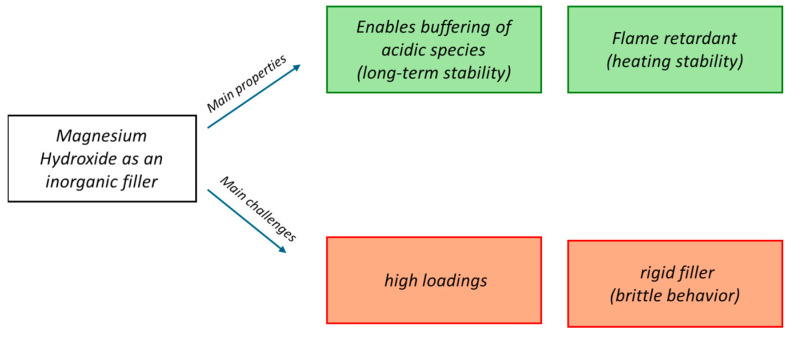
Pros and cons of MDH used as an inorganic filler in composites.

Second, its mild alkalinity enables the buffering of acidic species (such as carboxylic acids from polyester degradation or HCl from PVC or certain additives) improving long-term stability, reducing internal corrosion of metal components in contact with the polymer and moderating interactions with stabilizers and pigments [117]. Third, as a rigid inorganic filler, Mg(OH)_2_ modifies mechanical behavior: depending on particle size, morphology and surface treatment, it can increase stiffness and compressive strength, or, if poorly dispersed and used at very high loadings without compatibilization, induce brittle behavior. Modern approaches emphasize submicron or plate-like particles with appropriate organic or inorganic surface coatings, allowing relatively high filler contents while still retaining acceptable toughness and elongation in matrices ranging from polyolefins and elastomers to engineering plastics and biopolymers [118]. The high filler loadings required for strong flame retardancy inevitably alter melt rheology. In unmodified form, Mg(OH)_2_ can significantly increase melt viscosity, torque and die pressure during extrusion or molding, especially in cable and sheet compounds with high mineral content [119]. In the end, as shown in Figure 21, it is possible to summarize the main properties and main challenges of the use of MDH as a filler for eco-friendly composites, including: the exploitation of the flame retardancy properties without the release of any hazardous substances and the decay of the mechanical properties due to the high filler loading necessities.

### 4.1. Polyolefin Polymer Matrices

In bulk polymer applications, magnesium hydroxide is a consolidated halogen-free flame retardant for polyolefin matrices, particularly HDPE, LDPE/LLDPE (including XLPE) and PP [80]. In high-density polyethylene, several studies highlight the quantitative compromise between mechanical properties and Mg(OH)_2_ content. When HDPE is filled with 10–50 wt% Mg(OH)_2_, the flexural modulus and Young’s modulus increase steadily with filler fraction, while tensile fracture strength and tensile yield strength decrease as Mg(OH)_2_ content rises; tensile elongation at break drops sharply between 0 and 10 wt% and then decreases more gradually up to 50 wt%. In the same composites, compressive and shear strengths increase with Mg(OH)_2_ up to about 40 wt% and 30 wt%, respectively, reflecting the classical transition from ductile to mineral-dominated behavior as the inorganic phase becomes continuous or near-continuous. Similar HDPE/MH systems formulated specifically for flame retardancy typically require 40–60 wt% Mg(OH)_2_ to achieve self-extinguishing behavior, and one chapter on PE HD/MDH composites reports that 60 wt% Mg(OH)_2_ is necessary to reach the desired fire protection level [120]. In LDPE and LLDPE, and in XLPE for cable insulation, Mg(OH)_2_ is the reference metal hydroxide for low-smoke halogen-free cable compounds [121]. Crosslinked LDPE (prepared by silane grafting) filled with Mg(OH)_2_ has been studied in detail for cable insulation; a series of LDPE/MH formulations showed that increasing Mg(OH)_2_ loading improves LOI, reduces smoke density and enables the passing of flammability and aging tests, with a loading window identified where tensile strength, elongation at break, hot set, ozone resistance and impact performance still satisfy cable standards [122]. In LLDPE/MH blends, the use of grafted compatibilizers such as PE g dibutyl maleate (PE-g-DBM) significantly improves interfacial adhesion and thus mechanical behavior and morphology; suitable levels of PE g DBM in LLDPE/MH/compatibilizer blends increase tensile properties and produce a finer MH dispersion, although the overall crystallinity of the blends decreases with increasing compatibilizer content [123]. For LDPE-based cable systems, the incorporation of EVA and LDPE g MAH has been shown to improve the flowability and mechanical properties of LDPE/MH compounds, counteracting the strong viscosity increase and loss of elongation induced by ATH, MH or zinc borate at high filler contents; among the metal hydroxides, MH-containing formulations exhibit the strongest adhesion to the polymer, which is consistent with their better mechanical performance at equivalent loadings [124]. Together, these data delineate a typical design space in which LDPE/LLDPE/XLPE cable compounds carry 50–60 wt% Mg(OH)_2_, with tailored compatibilization and crosslinking sometimes needed to maintain tensile strengths in the order of 10–20 MPa and elongations at break above 100–150%, while achieving LOI ≥ 30% and acceptable smoke behavior [125].

In polypropylene, Mg(OH)_2_ plays a dual role as a flame retardant and as a structural/mineral filler in homopolymer and composite matrices, including WPC and natural-fiber-reinforced systems but, as discussed for polyolefins, mechanical properties deteriorate strongly if the filler is unmodified [126,127]. As summarized in Table 4, polypropylene-based systems have emerged as a powerful strategy to overcome the high loadings and mechanical penalties associated with Mg(OH)_2_ alone with the investigation of the synergistic combinations with titanium dioxide, layered and fibrous clays, natural fibers and wood phases in wood–plastic composites (WPCs), and graphite/expandable. A key example of synergism is provided by PP/Mg(OH)_2_/TiO_2_ systems, originally investigated to address the discoloration of PP compounds containing magnesium hydroxide [128,129]. In the study by Braun and colleagues on PP compounds filled with a commercial precipitated Mg(OH)_2_, the authors showed that compounding Mg(OH)_2_ with PP in the presence of phenolic antioxidants leads to coloration from light gray to dark beige, which is undesirable for many applications. They systematically replaced a small fraction of the Mg(OH)_2_ flame retardant with titanium dioxide and found that TiO_2_ not only restored a desirable white color but also acted as a true flame retardant synergist; replacing 1.5 wt% of FR with TiO_2_ significantly increased the LOI of the PP/MH formulation, with LOI values of the TiO_2_-containing compounds “much better than that of PP compounds containing Kisuma 5A or Magnifin H5C” at similar total filler content. In addition, dynamic thermal analysis showed that the thermal stability of PP was improved when part of the Mg(OH)_2_ was replaced by TiO_2_, with higher onset temperatures of degradation; this effect is particularly relevant for applications where the composite must withstand prolonged exposure to heat, oxygen and mechanical stress. Structurally, TiO_2_ contributes an inert, high-melting inorganic phase that cooperates with MgO residue in forming a rigid, opaque barrier, while Mg(OH)_2_ provides endothermic dehydration and basicity; the result is a material that is simultaneously better colored, more thermally stable and more flame-resistant than PP/MH alone at comparable mineral loading. Also, the synergistic action of Mg(OH)_2_ with clays in polypropylene has been evaluated in detail by Marosfői et al., who studied PP–Mg(OH)_2_–clay composites containing layer-like montmorillonite and needle-like sepiolite in both natural and organo-modified forms [130]. Fire performance was characterized by conical combustor and horizontal burning tests, while SEM and temperature-dependent rheology were used to probe the structure and mechanical strength of the combustion residue. The results show that fibrous and layered clay nanofillers can be combined advantageously with Mg(OH)_2_ microfillers: in PP/MH/clay systems, the time to ignition increases and the peak heat release rate decreases markedly compared with PP/MH without clay, leading to improvements in flammability classifications. For example, combinations of montmorillonite and sepiolite yielded the largest increase in time to ignition and the strongest reduction in heat release rate, attributed to a pronounced “char stabilizer” effect of the nanofillers. Rheological analysis of the residue at elevated temperatures indicated that the presence of clays significantly increases the stiffness of the molten/softened residue, consistent with the formation of a more robust mineral–carbon network in which MgO from Mg(OH)_2_ and silicate platelets/fibers interpenetrate. The authors concluded that not only micro–nanofiller interactions (between Mg(OH)_2_ and clays) but also nanofiller–nanofiller interactions among the different clays play a key role in controlling char strength and barrier efficiency, which in turn governs the overall flame retardant behavior of the PP/MH/clay composites. The combination of Mg(OH)_2_ with natural fibers in PP matrices provides another important synergy, in which flammability is reduced in fiber-rich composites while maintaining, to some extent, their mechanical advantages. Lee et al. investigated the mechanical and thermal properties of kenaf fiber reinforcing PP/Mg(OH)_2_ composites, focusing on kenaf fiber contents of 0–20 wt% and MH loadings tailored for flame retardancy [131]. Pure PP exhibits low tensile and flexural properties and poor flame behavior, whereas introducing kenaf fiber improves tensile modulus and mass residue at onset temperature, but reduces tensile strength, elongation at break and flexural strength due to the intrinsic brittleness of the fiber-reinforced structure. The incorporation of Mg(OH)_2_ further modifies the behavior: thermogravimetric analysis shows that the insertion of fiber and filler splits the thermal decomposition into two stages (a first step at lower temperature associated with fiber degradation and a second at higher temperature related to PP), and all composite samples display higher mass residue than neat PP, indicating that kenaf and MH cooperate to reinforce the char and shift degradation to higher temperatures. Although increasing MH content tends to reduce strength and elongation, the net effect is a system in which the natural fiber contributes stiffness and low density, while Mg(OH)_2_ raises thermal stability and fire resistance, making these composites attractive for semi-structural components where improved flame behavior is necessary. Other studies on natural fiber PP composites containing Mg(OH)_2_ (e.g., PP/flax/MH, PP/wood/MH) corroborate this synergistic trend. In PP/flax/MH systems with 30–50 wt% flax and up to 30 wt% Mg(OH)_2_, LOI values above 27 and very slow burning rates in horizontal tests are reported for high-fiber/high-MH formulations (e.g., 50 wt% flax/30 wt% MH), although no formulation attains vertical UL 94 classification due to the high inherent flammability of the fiber-rich matrix. In PP/wood/MH WPCs, the addition of Mg(OH)_2_ reduces the burning rate by about 50% in PP/sawdust and PP/rice husk composites relative to neat PP, while maintaining tensile and flexural moduli suitable for decking and outdoor profiles; the lignocellulosic phase and MgO residue jointly build a relatively rigid, thermally insulating char layer [132,133]. Within wood–plastic composites, Mg(OH)_2_ often acts as the primary halogen-free flame retardant, embedded in a complex microstructure comprising wood flour, coupling agents and sometimes additional nano- or microfillers. Recent work on PP-based WPCs emphasizes that metal hydroxides such as Mg(OH)_2_ can increase flame retardancy “without sacrificing mechanical properties” to the same extent as many halogenated systems, particularly when used in ultrafine form or in combination with layered hydroxides or nanoclays [132].

### 4.2. Other Systems of Polymer-MDH Composites

Flexible PVC and styrenic matrices such as PS and HIPS represent two important families of thermoplastics in which micro- and nanostructured magnesium hydroxide has been quantitatively shown to improve flame retardancy and smoke suppression, often within multi-component architectures that also preserve acceptable mechanical performance [117]. In flexible PVC, Mg(OH)_2_ behaves as an acid scavenger: the basic MgO surface partially neutralizes the HCl released during PVC dehydrochlorination, thereby reducing smoke acidity and corrosivity, as follows:

$$Mg{\left(OH\right)}_{2}\;\to MgO+{\;H}_{2}O$$

$$Mg{\left(OH\right)}_{2\;}+2HCl\to \;Mg{Cl}_{2}+2H_{2}O$$

A particularly illustrative example already discussed is the core@double shell microcapsule MH@DOPO@MF, in which a magnesium hydroxide core is coated with 9,10 dihydro 9 oxa 10 phosphaphenanthrene 10 oxide (DOPO) and an outer melamine–formaldehyde shell, providing an integrated Mg–P–N flame-retardant entity; this microcapsule was incorporated at 10 wt% into flexible PVC and compared with neat PVC and with PVC containing 20 wt% unmodified Mg(OH)_2_. The limiting oxygen index (LOI) of pure flexible PVC is reported as 21.9%, with no UL 94 rating, while PVC filled with 20 wt% unmodified Mg(OH)_2_ reaches only 23.8% LOI and still fails to achieve a UL 94 classification. By contrast, the PVC/10 wt% MH@DOPO@MF composite exhibits an LOI of about 30.9%, corresponding to a 9.1% absolute increase over neat PVC, and attains a UL 94 V 1 rating while keeping good mechanical performances [104].

In styrenic systems, Mg(OH)_2_ is used to tackle the inherently high flammability, high peak heat release rate and severe melt dripping behavior of PS and HIPS, typically in combination with other flame retardant phases to improve efficiency and reduce the total required inorganic loading. In PS-based formulations, Mg(OH)_2_ again acts through endothermic decomposition and formation of an MgO barrier, but its flame retardant performance is significantly enhanced when coupled with nanostructured co-fillers such as organo-modified montmorillonite (OMMT); PS/MH/OMMT nanocomposites reported in the literature exhibit reductions in peak heat release rate on the order of 30–50% and moderate increases in LOI compared with neat PS, while the combined effects of increased melt viscosity and the formation of an inorganic rich surface layer strongly diminish melt dripping in UL 94 tests [134,135]. For high-impact polystyrene (HIPS), more complex architectures such as HIPS/Mg(OH)_2_/microencapsulated red phosphorus (MRP)/glass fiber have been developed to simultaneously enhance flame retardancy, control thermal conductivity and preserve adequate impact resistance for structural and housing applications in electrical and electronic equipment. In these HIPS/MH/MRP/GF composites, Mg(OH)_2_ serves as an inorganic flame retardant and smoke suppressant, MRP provides a phosphorus-rich phase that promotes char formation, and a small fraction of glass fiber (around 2 wt%) forms a reinforcing network that both increases stiffness and supports the char skeleton during combustion, thereby stabilizing the specimen and strongly reducing the emission of flaming drips that are typical of unfilled or solely Mg(OH)_2_-filled HIPS [136]. Fire testing and thermal conduction measurements on such systems show that the introduction of glass fiber increases the effective thermal conductivity of the composite while improving UL 94 performance by inhibiting melt dripping and facilitating rapid self-extinguishment; at the same time, increasing the combined Mg(OH)_2_/MRP content lowers peak heat release rate and burning rate relative to neat HIPS or HIPS containing only Mg(OH)_2_. Although higher mineral and glass contents inevitably raise the elastic modulus and reduce elongation at break, the HIPS matrix retains sufficient impact strength to remain suitable for molded housings and structural parts, so that HIPS/MH/MRP/GF formulations emerge as an example of how magnesium hydroxide can be integrated into multi-component flame-retardant architectures to tune UL 94 rating, dripping behavior and thermal conductivity, enabling the design of flame-retardant, thermally conductive polymer components where heat dissipation, mechanical robustness and fire safety requirements must all be satisfied [136].

Therefore, pioneering studies investigated the formulation of high-impact polystyrene (HIPS)-based composites containing 40–50 wt.% of Mg(OH)_2_ and Mg(OH)_2_ modified with triblock copolymer styrene/butadiene/styrene (SBS) to successfully formulate materials with medium smoke suppression properties which can be used to produce nearly all elements of rolling stock equipment. The modification of Mg(OH)_2_ with SBS leads to the formulation of HIPS-based systems with improved mechanical properties, more specifically, with improved elongation ability and ductility [137,138].

Poly(ethylene terephthalate) (PET) has been rendered highly flame retardant by combining it with magnesium-based hybrid systems. In one approach, carbon microspheres coated with magnesium hydroxide (Mg(OH)22@CMSs) are synthesized and characterized by SEM and FTIR, then melt-blended into PET at very low loading; with only 1 wt% Mg(OH)22@CMSs; the composite achieves a limiting oxygen index (LOI) of 27.5% and attains a UL 94 V 0 rating, indicating a strong suppression of flammability compared with neat PET (LOI ≈ 21%, typically no V 0 rating). Cone calorimeter measurements and thermogravimetric analysis, supported by SEM and FTIR of the char residues, demonstrate a clear synergistic action between Mg(OH)22 and the carbon microspheres: during combustion, Mg(OH)22 decomposes together with the CMSs and forms dispersed “bridge” supporting points within the evolving carbonaceous residue, which reinforce a continuous, compact three-dimensional char network that reduces both the peak heat release rate (pk HRR) and peak mass loss rate (pk MLR). At the same time, FTIR spectra of the Mg(OH)22@CMSs/PET char show an increased presence of unsaturated species with C=C bonds, implying that the hybrid filler induces PET to degrade into smaller unsaturated fragments that are more prone to crosslinking and char formation, thus increasing the solid residue and decreasing the total heat release (THR) compared with unfilled PET [139]. A related strategy employs magnesium–aluminum layered double hydroxides (MgAl LDHs) that are modified and then incorporated into PET by in situ polymerization, so that the inorganic phase participates directly in chain growth. In this system, MgAl LDHs with different particle sizes are first modified with Sulfanilic Acid Salt (SAS) by a hydrothermal process to obtain MgAl LDH SAS; the organic anions improve compatibility with the PET precursor, and the LDH is then intercalated with bis hydroxy ethylene terephthalate (BHET) before being used as both catalyst and nanofiller in PET polycondensation. The presence of MgAl LDH SAS increases the polymerization rate without causing premature decomposition of PET, and the modified galleries facilitate the penetration of oligomers, so that polymer chains can grow between the LDH layers, yielding intercalated or partially exfoliated PET/MgAl LDH SAS nanocomposites. As a result of this architecture, the materials exhibit improved gas barrier performance, enhanced thermomechanical properties, and modified crystallization behavior, all of which are favorable attributes for flame-retardant PET systems where reduced oxygen and fuel diffusion, together with tailored crystallinity, contribute to lower flammability and better dimensional stability under heat [140]. To further optimize the PET/filler interface, magnesium hydroxide has been employed as a capsule wall on carbon microspheres, followed by organic functionalization, to create double layer particles that couple efficient flame retardant action with strong interfacial bonding. In this design, Mg(OH)22 is deposited on the surface of CMSs by liquid-phase deposition to obtain MH@CMSs, which are then modified with 3 aminopropyltriethoxysilane (APTS) to form FMH@CMSs; FESEM, TEM, FTIR, and XPS confirm the formation of an inner inorganic Mg(OH)22 shell and an outer organic silane layer, while TGA reveals the coating degree and improved thermal stability. PET composites prepared by melt-compounding with MH@CMSs or FMH@CMSs show that the silane modification significantly strengthens the interfacial binding forces, leading to markedly better stress transfer and less embrittlement compared with the unmodified core–shell filler. At 1 wt% FMH@CMSs, the LOI of PET increases from 21% to 27.6% and UL 94 V 0 is achieved, similarly to the Mg(OH)22@CMSs system, but, crucially, the tensile strength of the composite rises by 66.2% to 47.20 MPa, a value nearly identical to that of neat PET, demonstrating that the two-layer structure (Mg(OH)22 capsule wall plus APTS-based organic shell) allows the simultaneous realization of high flame retardancy and retention of the intrinsic mechanical performance of PET. Collectively, these studies show that PET can act not only as a structural thermoplastic matrix, but also as a versatile platform for advanced magnesium-based flame-retardant architectures—ranging from Mg(OH)22/carbon core–shell particles to LDH-based nanocomposites and epoxy/PET fiber hybrids—in which low filler loadings around 1 wt% are sufficient to reach LOI values near 27–28% and UL 94 V 0, provided that the filler morphology, surface chemistry, and dispersion are carefully engineered [141].

In another study, POM copolymer M90 (another widely used thermoplastic) was compounded with magnesium hydroxide (MH) as the principal inorganic halogen-free flame retardant and a series of organic synergists, namely melamine (ME), linear novolac resin (LNR) and triphenyl phosphate (TPP), with the dual aim of improving flame retardancy and controlling the gas-phase products of thermal degradation. MH was selected because of its endothermic dehydration and formation of MgO, as well as its basic nature, which can neutralize formic acid, whereas ME, LNR and TPP were designed to act as formaldehyde/formic acid scavengers and condensed-phase char promoters. The formulations were systematically varied, and the UL 94 ratings, LOI, thermal analysis and characterization of gaseous and condensed-phase residues were used to elucidate the synergistic mechanisms [142]. Other studies investigated the introduction of benzoxazine monomers of traditional bisphenol-A benzoxazine (BA-a) and bisphenol-A benzoxazine containing trialkoxysilane (BA-a-Si) synthesized and incorporated into polyoxymethylene/ammonium polyphosphate/melamine (POM/APP/ME) to improve the fire retardancy of POM. The POM-based materials were investigated for their flame retardation, thermal, and mechanical properties that were evaluated by cone calorimeter, limiting oxygen index (LOI), and the Underwriters Laboratories-94 (UL-94) vertical burning tests, as well as thermogravimetric analysis and mechanical tests, respectively. However, the benzoxazine, especially siloxane-containing benzoxazine, played an effective role in flame-retarding POM acting as a charring agent and synergistic flame retardant [143,144].

Acrylonitrile–butadiene–styrene (ABS) is a widely used engineering thermoplastic used in 3D-printing technologies but shows high flammability, with typical limiting oxygen index (LOI) values around 18–19 vol.% and rapid heat release under external heat flux, which severely restricts its use in fire-sensitive applications. In ABS composites filled with magnesium hydroxide sulfate hydrate (MHSH) whiskers, prepared by melt-compounding, increasing MHSH content (e.g., 5–20 wt.%) leads to a substantial reduction in average heat release rate (HRR) and peak HRR in cone or microcone calorimetry compared with neat ABS, whose average HRR can reach about 260–270 kW m^−2^. The incorporation of zinc stearate as a processing/dispersing aid improves MHSH dispersion, as confirmed by scanning electron microscopy (SEM), and further lowers HRR and mass loss rate (MLR), indicating that better distribution and interfacial wetting of the whiskers enhance the flame retardant efficiency at a given loading. SEM analyses of char residues show that ABS/MHSH systems retain a fibrous or network-like morphology after combustion, consistent with a reinforcing mineral skeleton that stabilizes the condensed phase and contributes to barrier effects against heat and mass transfer. Thermogravimetric analysis (TGA) reveals that MHSH (and MH) raises the onset of major mass loss and increases the temperature at maximum degradation rate, often by tens of degrees Celsius relative to pristine ABS, due to the endothermic decomposition of the hydrated phase and the protective MgO layer formed at higher temperatures [145,146,147]. From a rheological perspective, the viscoelastic behavior of ABS/MHSH composites is significantly modified at low frequencies, where the complex viscosity and storage modulus can increase by roughly one order of magnitude when the whisker content is raised from 0 to about 20–30 wt.%, and the materials exhibit a more pronounced solid-like response in the terminal region than unfilled ABS, reflecting the formation of a weakly percolated filler network. Mechanical tests on ABS filled with surface-modified MH whiskers show that the elastic modulus increases from values typical of neat ABS (≈1.5–2.0 GPa) to about 2.2–2.4 GPa at intermediate loadings, with one study reporting an elastic modulus of 0.16 GPa (test specific value) and a tensile strength of 20.1 MPa and total elongation at break of 10.2% at 10 wt.% whiskers, identified as the composition with the best overall balance of properties. At higher MH loadings (≈20–25 wt.% and above), the modulus tends to plateau or reach a maximum, whereas tensile strength and elongation at break decrease progressively as the inorganic fraction increases, highlighting the need to optimize interfacial adhesion and whisker geometry to balance stiffness and toughness [148]. Nevertheless, when MH is combined with additional fillers and processing strategies—such as microcellular foaming using supercritical CO_2_ and the co-dispersion of MH with nanoclay—ABS/MH/nanoclay foams with relative densities around 0.9–0.95 can achieve reduced polymer consumption without significant deterioration in yield and tensile strength or impact resistance, and in some cases even show modest increases in modulus relative to solid counterparts at similar filler loadings, while maintaining thermal properties such as heat deflection temperature and Vicat softening point within the same order of magnitude as unfoamed ABS. In such microcellular systems, the refined cell size (often in the micrometric or sub-micrometric range) and the presence of well dispersed nanoclay/MH domains contribute to the mechanical stabilization of the foam structure, so that dimensional integrity during injection molding is preserved despite the lower polymer content. Taken together, these quantitative data demonstrate that MH and MHSH whiskers, especially when properly dispersed and combined with synergistic fillers or foaming technologies, provide an effective halogen-free pathway to improve the flame retardancy and thermal stability of ABS, while preserving acceptable mechanical performance and, at optimized loadings (~10–25 wt.% MH), even enabling tailored stiffness–toughness trade-offs suitable for engineering applications [149].

### 4.3. Expandable Polyurethane Matrices

Magnesium hydroxide (MH) has emerged as a highly versatile filler also for polyurethane (PU) systems in thermal applications, where flame retardancy must often be combined with mechanical integrity, surface functionality, and specifically in the automotive sector noise, vibration, and harshness (NVH) control. In PU foams the incorporation of MH not only alters the acoustic response through microstructural control but also contributes to heat dissipation and improved fire behavior, while in coatings and fibers MH enhances char formation, reduces heat release, and enables multifunctional thermal–mechanical–biological performance [150]. In flexible or semi-rigid PU foams used for automotive NVH control, MH has been introduced primarily to tailor open-cell morphology and thus the sound absorption behavior, but the same microstructural features are intrinsically linked to thermal transport and thermal stability [151]. In PU composite foams containing magnesium hydroxide as a particulate filler, the noise reduction coefficient (NRC) increases by about 70% at an optimum open porosity of 0.63 relative to the non-filled foam, with the best performance at only 1.0 wt% MH, highlighting a strong structure–property relationship between a small filler loading and a large gain in acoustic efficiency. This improvement is attributed to two main mechanisms:

(i) Enhanced damping due to the rigid MH inclusions embedded in the viscoelastic PU matrix;
(ii) An increased number of partially opened cells and well-developed cavities, which intensify viscous and thermal losses of sound waves within the pore network.

This optimized microstructure with finer cells and interconnected pores promotes more homogeneous heat distribution and can slow down flame spread because the tortuous gas paths and increased internal surface area favor heat absorption and gas-phase dilution when MH decomposes endothermically above about 300 °C, releasing water vapor and forming a protective MgO-rich layer. In rigid PU foam laminates manufactured from isocyanate, polyether polyol, 10 wt% flame retardant, and combinations of aluminum hydroxide (ATH) and MH at 0–20 wt%, the foam core is integrated with nylon nonwoven fabrics and polyester–aluminum foil to form structural panels whose cell structure, compressive stress, combustion resistance, thermal stability, sound absorption, and electromagnetic interference shielding effectiveness (EMI SE) were systematically evaluated. Although MH contributes to foam integrity and thermal protection, aluminum hydroxide exhibits superior overall performance in this specific system; at 20 wt% ATH the composite reaches an optimal density of 0.153 g/cm^3^, an average cell size of 0.2466 mm, a maximum compressive stress of 546.44 kPa, an LOI of 29.5%, and an EMI SE of 40 dB, together with excellent thermal stability and sound absorption. This comparison indicates that while MH is particularly efficient at low loadings for tuning porosity and providing halogen-free flame retardancy, ATH can deliver higher LOI and compressive strength at higher concentrations, which is crucial for thermally robust structural acoustic panels. Nevertheless, MH remains attractive where the balance between acoustic damping, foam processability, and moderate flame retardancy is more critical than maximizing LOI alone, especially considering its lower decomposition temperature relative to ATH and its potential synergistic effects with other flame-retardant additives in PU matrices [150,151,152,153,154].

### 4.4. Polyamide for Packaging Purposes

Magnesium hydroxide (MH, Mg(OH)_2_) has emerged as a premier halogen-free flame retardant for polyamide (PA) matrices, including PA6, PA66, PA11, and their copolymers, with a focus on their implications for sustainable PA-based packaging materials, where FR, lightweighting, barrier properties, and recyclability are paramount for food, electronics, and industrial containers. A representative study on PA6 shows that a methyl-blocked novolac (MBN), synthesized via a Williamson ether route, acts as a multifunctional char former and compatibilizer when combined with MH. Blocking the phenolic hydroxyl groups in the novolac reduces their susceptibility to thermo-oxidative degradation and enhances the thermal stability of the additive compared with the unmodified novolac. In PA6, MBN markedly promotes char formation and suppresses melt dripping during burning, enabling a stable, cohesive carbonaceous layer that improves flame retardancy. At the same time, MBN behaves as an efficient lubricant and interfacial agent between MH and PA6, improving processability (lower melt torque, higher melt flow) and leading to finer MH dispersion in the matrix; as a result, flame retardant PA6 with a good combination of mechanical and processing properties can be obtained, which is vital for extrusion and film/blow molding operations in packaging [155]. The use of MH produced via magnesia hydration followed by jet milling has been demonstrated as an effective flame retardant in a nylon 6–6,6 copolymer, which is directly relevant to high-performance packaging where PA66 or PA6/66 are often used for thermoformed trays and barrier layers. In this process, MgO is hydrated in an autoclave at 130 °C for 1 h, and the resulting MH is further comminuted in a jet mill to obtain a controlled particle size distribution. When compounded into a nylon 6–6,6 matrix, the hydrated MH provided a V 0 UL94 rating at 1.6 mm with 60 wt% MH, and at 3.2 mm with 40 wt% MH, while maintaining mechanical properties at acceptable levels for structural applications. These results indicate that MH obtained from magnesia hydration can be successfully employed as a fire retardant for nylon-based systems, and that its controlled particle size and surface area are key levers to balance flame retardancy and mechanical performance in packaging components that must withstand handling, filling, and sealing operations [156]. To tackle the poor processability typically observed in highly filled PA6/MH systems, a novel processing method—solid-state shear milling (S3M)—has been introduced, using specially designed pan mill equipment to compound high loadings of inorganic MH with PA6. In this approach, PA6 and MH are co-milled in the solid state, which effectively pulverizes PA6, increases the interfacial interaction between the resin and MH, and achieves highly uniform blending at the solid state, thereby controlling the state of the dispersed phase before melt-processing. The co-milled composite powder serves as a flame retardant masterbatch that is subsequently melt-blended with neat PA6 pellets; this strategy greatly improves the compatibility of the system, modifies the distribution process of MH during melt-compounding, and reduces the dispersion-phase size in the final melt-processed material. As a result, S3M technology significantly increases the melt flowability of the composites and leads to clearly enhanced flame retardancy and mechanical performance compared with direct melt-processing, offering an effective solution to the typical processing difficulties and property deterioration seen in conventionally compounded PA6/MH systems. For packaging, this implies that high MH formulations can still be processed into thin films, multilayer structures, or injection molded containers without sacrificing throughput or surface quality, which are critical for industrial scaling [157,158]. The use of MH as a flame retardant in PA packaging can be combined with other functionalities such as smoke suppression, mechanical reinforcement, or tribological performance, extending the application range beyond simple FR compliance. Work on MH-filled PA blends has shown that appropriate formulation can maintain or even improve impact strength and toughness despite high inorganic loadings, which is highly desirable for rigid packaging that must resist drop impacts and mechanical abuse during logistics. Studies on MH in polyamide coatings produced by plasma spraying indicate that MH dehydrates to MgO in a similar temperature range as PA degradation, and that the extent of dehydration depends on particle size, allowing some control over the amount of unreacted MH retained in the coating. Although the suppression of matrix degradation was not dramatically improved in those coatings, the ability to retain a high content of unreacted MH in plasma-sprayed PA composites suggests potential for engineering applications where both wear resistance and fire retardance are relevant, such as protective PA-based coatings on packaging equipment or reusable containers exposed to friction and heat [159,160]. Tribological studies of PA66 composites containing 20 vol% MH have shown that local flash temperatures at sliding interfaces are sufficient to trigger the endothermic decomposition of MH, and that the resulting reaction products accumulate within the transfer film on steel counterparts, leading to reduced friction and wear. This concept of MH as a tribologically active filler indicates that PA/MH materials might not only impart fire safety to packaging but also improve durability in moving or reclosable packaging systems (e.g., threaded closures, slide mechanisms), potentially reducing material consumption over the life cycle. In the broader context of eco-design for packaging, these MH-based systems align with halogen-free, low-smoke, and recyclable material strategies, particularly when combined with bio-based polyamides or multilayer structures where the PA/MH layer acts as both a structural and a safety enhancing element. From a packaging standpoint, MH-filled PA6 and PA66 systems synergized with organic char formers like MBN or processed via S3M offer a pathway to thin-walled articles and films that meet high fire safety standards (e.g., UL94 V 0 at relevant thickness) while retaining the toughness, processability, and surface quality demanded by food, cosmetic, or electronics packaging. The tunability of MH particle size and morphology via magnesia hydration and jet milling, combined with compatibilization strategies, allows the design of formulations with controlled rheology and reduced filler-induced defects, which is crucial for high-speed film extrusion, stretch blow molding, and thermoforming lines. Moreover, the inherent low smoke and low toxicity of MH-based systems compared with halogenated flame retardants addresses consumer safety concerns and facilitates compliance with emerging fire safety requirements for transport and storage packaging in confined environments. In this way, MH-based polyamide systems provide a versatile platform for next-generation flame-retardant packaging that combines regulatory compliance, functional performance, and environmental responsibility.

### 4.5. Elastomer Matrices

Magnesium hydroxide-based systems in SEBS thermoplastic elastomers offer an effective, fully halogen-free route to combine flame retardancy with acceptable elasticity, especially when MH is used together with aluminum hydroxide or with tailored interfacial compatibilization (MA SEBS, silane-treated MH). EBS-based composites plasticized with paraffinic extender oil (O SEBS), blended with PP and filled with ATH or MH, show a strong increase in LOI with increasing hydroxide loading. At a given filler content, ATH produces higher LOI than MH, indicating somewhat superior flame-retardant efficiency in this matrix; for example, at high total load (around 65 wt.% ATH + MH), LOI rises markedly compared with the unfilled blend, and the ATH-only system reaches LOI values clearly above those of the MH-only counterpart. When MH is progressively introduced into ATH-filled composites at constant total filler content of 65 wt.%, LOI remains nearly unchanged or slightly enhanced until the MH/ATH ratio exceeds about 40%, and only then begins to decrease. This behavior suggests a synergistic effect between ATH and MH, since mixed systems maintain high LOI even though part of the more efficient ATH is replaced by MH. Both hydroxides also improve thermal stability; TGA/DTG curves show delayed onset of mass loss and higher residual mass for filled SEBS-based materials, with MH giving a somewhat larger stability increase than ATH, which is valuable for applications requiring higher service temperatures. Mechanically, tensile strength and elongation at break decrease as filler loading increases up to 50 wt.%, reflecting the usual stiffening and embrittlement associated with high-mineral content. However, at a fixed loading the composites filled with MH retain slightly higher elongation at break than those with ATH, while tensile strength is similar for both fillers, making MH attractive where some elasticity must be preserved. At very high filler contents above 50 wt.%, tensile strength levels off whereas elongation drops sharply, delimiting the practical upper bound for highly filled flame-retardant SEBS formulations. For a combined 65 wt.% total ATH + MH, varying the MH fraction markedly influences ductility; the elongation at break increases from about 180% to 200% as the MH/ATH ratio is raised to 40%, then jumps to roughly 300% when MH reaches 55–60 wt.% of the total hydroxide, before decreasing again at higher MH contents. This non-monotonic trend has been attributed to differences in particle size distribution between ATH and MH; an appropriate balance of fine and coarse particles can optimize stress transfer and reduce defect sensitivity in the elastomeric matrix [161]. A second study on SEBS/PP/O SEBS composites compares untreated MH with silane coupling agent-treated MH (m MH) and explores the effect of partially replacing oil-filled SEBS with maleic-anhydride-grafted SEBS (MA SEBS). As MH or m MH content increases, flame retardancy improves (higher LOI, better burning behavior), but tensile strength and elongation at break both decrease, and melt flow deteriorates due to the high filler level and increased melt viscosity. Replacing part of the O SEBS with MA SEBS has a clear quantitative impact on mechanical performance. At a given MH content, the introduction of MA SEBS increases tensile strength, while elongation at break decreases, consistent with stronger interfacial bonding and a more rigid network. When MA SEBS is combined with silane-treated MH (m MH), the improvement is more pronounced; tensile strength rises by a “large margin” compared with systems containing only O SEBS and untreated MH, demonstrating the effectiveness of chemical coupling at the polymer–filler interface. SEM analysis of fracture surfaces confirms that MA SEBS enhances filler–matrix adhesion, showing fewer voids and better wetting of MH particles, especially for m MH. TGA data reveal that both MH and m MH increase the thermal stability of SEBS-based composites, with the modified filler offering slightly more favorable decomposition behavior, which supports improved fire resistance and durability at elevated temperature. These interfacial design strategies are particularly relevant for high filler flame-retardant elastomeric parts such as cable sheathing, flexible connectors and gaskets, where the retention of mechanical integrity is as important as meeting fire standards [162].

### 4.6. Magnesium Hydroxide Action for Degradation in Biopolymers

Magnesium hydroxide-based systems in PLA and PLLA offer a versatile toolbox to couple flame retardancy with controlled degradation and bio-functionality, especially when MH is surface-modified or combined with other inorganic/organic structures. PLA filled with stearic-acid-modified MH (mMH) from seawater shows that surface chemistry and dispersion strongly control both thermal behavior and degradation. FT IR confirms chemically bonded stearate on MH, and XRD plus FT IR selection leads to a high SA modified grade (m10MH) used in melt-mixed PLA/m10MH composites. DSC reveals that crystallization is primarily governed by the filler; PLA/m10MH composites crystallize differently from neat PLA, but XRD and XµCT show that increasing m10MH content actually decreases PLA crystallinity while increasing porosity and promoting mMH agglomeration. Thermogravimetric analysis shows that PLA/m10MH composites degrade in four distinct stages, and as m10MH loading rises the degradation pattern becomes more complex and overall thermal stability worsens, indicating that this specific modification does not act as a classical flame-retardant system. From a fire safety perspective, the increased porosity and agglomeration may even penalize performance, so this route is more illustrative of the limits of poorly optimized MH treatments in PLA than of a robust FR strategy [163], although it could be very interesting to study the degradation of this biodegradable matrix. Across these PLA family systems, MH and MH-derived structures act through intertwined mechanisms that are highly relevant for degradation:

PLA and PLLA degrade by water uptake, ester hydrolysis and diffusion/accumulation of lactic-acid-type products, which locally lower pH and accelerate chain scission (backbiting and transesterification). In neat PLLA and PLLA/PLCL blends without Mg(OH)_2_, molecular weight can drop to about 10% of the initial value within 14 days in aggressive in vitro conditions, showing very fast degradation once acid autocatalysis starts. The acidic medium not only speeds up bulk degradation but also drives intense inflammatory responses in vivo, which is a major problem for vascular devices and other implants [163,164,165]. PLA and PLLA degrade by water uptake, ester hydrolysis and diffusion/accumulation of lactic-acid-type products, which locally lower pH and accelerate chain scission (backbiting and transesterification). In neat PLLA and PLLA/PLCL blends without Mg(OH)_2_, molecular weight can drop to about 10% of the initial value within 14 days in aggressive in vitro conditions, showing very fast degradation once acid autocatalysis starts. The acidic medium not only speeds up bulk degradation but also drives intense inflammatory responses in vivo, which is a major problem for vascular devices and other implants. Mg(OH)_2_ is a sparingly soluble base; in contact with water and acids it consumes protons and forms Mg^2+^ and water, thus buffering the microenvironment. In PLLA/PLCL blends, Mg(OH)_2_ particles neutralize acidic degradation products, hindering their accumulation and preventing the strong pH drop normally seen in PLLA-only systems. This buffering slows the autocatalytic hydrolysis, so molecular weight decreases much more slowly; in PLLA100/Mg5, degradation is “scarcely” progressed over 14 days compared with almost complete degradation of PLLA without Mg(OH)_2_. By binding to carboxyl end groups and acidic oligomers, Mg(OH)_2_ suppresses backbiting and intermolecular transesterification, which are key pathways for rapid chain scission in PLA/PLCL. As a result, Mg(OH)_2_-containing blends retain mechanical strength longer during degradation, showing less embrittlement and better load-bearing capacity over time. In systems where MH is surface-modified with oligolactide (e.g., Mg OLA), the neutralizing effect is maintained, while dispersion and interfacial adhesion improve, leading to simultaneous reinforcement and better biological response [164,165,166].

On this view, other biopolymers can be studied for more industrial applications such as PBS. In PBS, magnesium-based fillers can either accelerate biodegradation (under marine conditions) or act as efficient flame retardants and reinforcements, depending on their chemistry, morphology and particle size [163]. Stearate-modified Mg–Al LDH (St Mg Al LDH) dispersed in PBS accelerates degradation in seawater, making these composites interesting where controlled marine biodegradation is desired rather than flame resistance. Melt-processed PBS/St Mg Al LDH films with 5–10 wt% LDH show clear surface erosion after 5 weeks and substantial disintegration after 10 weeks of immersion, whereas neat PBS is known to degrade very slowly in marine conditions. SEM reveals extensive surface cracking and pits in the LDH-filled films and dense colonization by diatoms and other microorganisms in the vicinity of degraded zones, indicating that LDH promotes both hydrolytic and bio-induced degradation. Thermally, the composites display lower onset degradation temperatures than pure PBS, showing that Mg and Al in the LDH catalyze PBS chain scission rather than stabilizing it. This catalytic effect is consistent with Lewis acid/Lewis base sites on LDH layers and with carboxylate–metal interactions facilitating ester bond cleavage. Mechanical and DMA tests, however, indicate that at the loadings studied the LDH does not significantly deteriorate tensile properties, so an accelerated marine degradation profile is achieved without strong loss in initial stiffness or strength. From an industrial point of view, such systems target degradable marine packaging or aquaculture films rather than classical flame-retardant PBS [167,168].

In conclusion, the versatility of magnesium hydroxide as a halogen-free flame retardant is unequivocally demonstrated across a diverse array of polymer matrices, as evidenced by the reviewed literature. Table 5 summarizes key applications, highlighting MDH’s adaptability in both commodity and engineering polymers for high-value, fire-safety-critical products. MDH excels in polyolefins such as HDPE for wood–polymer composites (WPCs) and low-smoke extruded profiles like decking and cable jackets [119,120]; LDPE/LLDPE for flexible, halogen-free cable sheathing and building membranes [121,122,123,124,125]; and PP for flame-retardant WPCs, structural profiles, and molded parts in fire-prone settings [126,127,128,129,130,131,132]. In halogen-sensitive contexts, it enhances PVC rigid profiles and low-smoke cable jackets, often synergizing with or partially replacing Al(OH)_3_. Styrenics like PS benefit in reduced-flammability sheets, panels, and decorative elements [135,136], while PET enables flame-retardant fibers, films, and recycled construction panels [137,138,139]. Engineering thermoplastics further underscore MDH’s broad utility: POM precision components; ABS halogen-free housings, automotive parts, and 3D-printable profiles targeting UL 94 V-0/V-1 [141,142,143,144,145]; PA textiles and electrical connectors with self-extinguishing properties [151,152,153,154,155,156]; and SEBS elastomers for soft fire-protective coverings [157,158]. Another interesting application is related to the formulation of sheathing for cables based on EVA and containing MDH [159]. Even in sustainable matrices, MH imparts efficacy to PLA biocomposites for panels and 3D printing [160,161,162,163] and PBS biodegradable films/packaging [164,165]. Polyurethanes (PU) foams and coatings round out the spectrum with lowered burn rates [146,147,148,149,150].

Therefore, Table 6 summarizes the main mechanical properties, density, and melt flow/processability of polymer and biopolymer composites containing MDH at a high loading level, based on the available literature. Overall, adding MDH at high loadings significantly increases the stiffness/elastic modulus of all types of composites. However, the elongation at break decreases drastically, leading to a complete loss of ductility and resulting in a totally brittle material. The decrease in tensile and impact strength values can be explained by poor stress transfer and MDH particle agglomerations, creating weak points. As expected, the addition of MDH increases the hardness, density and heat distortion resistance because the inorganic particles restrict polymer chain mobility. The restricted mobility of polymer chains in the melt state due to the presence of MDH particles could be considered responsible for the reduced processability and melt flow ability of polymer and biopolymer composites. This could be partially corrected by introducing plasticizers, coupling agents and/or modifying the inorganic particles, as discussed above.

## 5. Conclusions and Future Perspective

The analysis carried out on Mg(OH)_2_-filled systems across a broad range of thermoplastic and bio-based matrices shows that this inorganic filler can provide an effective, halogen-free route to flame retardancy in wood and natural fiber composites, cable compounds and structural profiles, while maintaining acceptable mechanical performance when properly dispersed and compatibilized. Nevertheless, the high loadings typically required, together with processing and toughness penalties, remain the main bottlenecks for large-scale adoption in demanding applications such as structural WPCs and high-performance engineering plastics. Overall, the compiled literature confirms that Mg(OH)_2_ is particularly attractive for HDPE, LDPE/LLDPE and EVA in low-smoke halogen-free cable sheathing, as well as for PP-based WPCs and selected biopolymers (PLA, PBS) in building and interior products where environmental compatibility and low smoke toxicity are key drivers.

Future research should prioritize surface-engineered and nanostructured magnesium hydroxide (Mg(OH)_2_) grades that maximize fire performance at reduced filler contents through tailored morphology, specific surface area, and interfacial chemistry with both polyolefin and polar matrices. In parallel, synergistic systems combining Mg(OH)_2_ with phosphorus-, nitrogen- or silicon-based components (including bio-derived species, such as phytic acid hybrids) appear essential to decreasing the peak heat release rate and smoke further, while mitigating losses in toughness and processability. Particular attention should also be given to Mg(OH)_2_-containing wood–plastic composites (WPCs) based on recycled or bio-based polymers. Optimizing filler treatment, coupling agents and processing conditions could support circular economy targets and extend the use of these materials to exterior building products subject to stringent fire codes. Finally, future work must increasingly link advanced flammability testing (cone calorimetry, smoke toxicity and large-scale fire scenarios) with durability, weathering and life cycle assessment to establish Mg(OH)_2_-based, halogen-free flame-retardant composite materials as robust, safe and sustainable solutions for the next generation of building, cable, transportation and bio-based material applications.

Current legislation in EU countries is gradually phasing out halogen-based fire retardants in favor of naturally occurring, halogen-free alternatives. Even in the event of an accidental fire, these next-generation retardants do not release harmful gases. Instead, they release water molecules that extinguish the flames and form protective layers that prevent the fire from spreading. It can certainly be asserted that magnesium hydroxide is one of the most important fire retardants that can be added to a variety of polymer and biopolymer matrices. Overall, it should be noted that adding high amounts of magnesium hydroxide can improve stiffness, hardness and heat distortion resistance, but this reduces ductility and processability. This can be corrected by using plasticizers, coupling agents, etc., or by modifying the surface of the inorganic particles.

## Figures and Tables

**Table 1:** Summary of product prices, productivity, scale-up complexity and final product quality of MDH powders.

	Costs	Productivity	Scale-Up Complexity	Final Product Quality
**Magnifin process**	Product is sold at least €2/kg for premium quality	Less than 5000 tons/years	Already industrially adopted	Mean particle size 0.3–1.3 μm; Specific surface area 1–5 m^2^ g^−1^; purity > 98.8%
**Acid leaching/alkaline precipitation process**	€1.5/kg up to €3/kg depending on purity and washing efficiency	More than 5000 tons/year	Already industrially adopted, such as the Aman process	Purity 99.3% (Aman process utilized by Dead Sea Periclase Ltd., Mishor Rotem, Israel)
**From magnesite via calcination–hydration**	€0.5/kg to around €1.5/kg for big bulk due to large disponibility	Around 20,000 tons/year (the most industrial mature process)	Already industrially adopted	Purity strongly related to magnesite source usually porous structure with impurities
**Dolomite use**	€1/kg to €2/kg, intermediate cost due to the separation of Ca/Mg	More than 5000 tons/year	Adopted industrially by Martin Marietta Magnesia Specialties (MagShield suitable for flame retardant applications)	Purity strongly related to dolomite source
**Saltwork bittern**	Optimized Mg(OH)_2_ cost is <€2/kg at pilot scale and <€1/kg at industrial scale	Expected more than 5000 tons/year	Require a post-treatment of crystals to meet flame retardant standards	>98.8% purity; surface area >20 m^2^/g; PSDs 1–10 micrometers

**Table 2:** A summary of promising advantages for the discussed innovative methods.

	Microwaves	Ultrasounds	Microemulsions
Main role	Rapid volumetric heating	Cavitation, intense mixing	Nanoscale confinement of reactants
Typical morphology	Nanoplates, fibers, nanosheets	Finer, less agglomerated crystals	Core–shell, highly uniform nanoparticles
Process advantage	Strong time reduction, low T	Better size control in bulk systems	Precise size and interface engineering

**Table 3:** Operational conditions of different fatty acid treatments.

			Ultrasound Stirring Optional Conditions			Hydrophobicity
**n:**			**Dosage**	**Temperature**	**Time**	**Contact Angle**
**1**	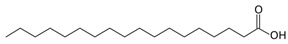	Stearic acid	1.25 wt%	70 °C	10 min	109.5°
**2**	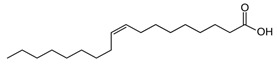	Oleic acid	7 wt%	60 °C	3 h	106°

**Table 4:** State-of-the-art PP-MDH FR composites.

Material System	Purpose of the Work	References
PP + 50 wt% Mg(OH)_2_ (unmodified MH)	enhance FR properties (low mechanical performances)	[126,127]
PP + Mg(OH)_2_ with partial replacement by TiO_2_ (e.g., 48.5 MDH + 1.5 TiO_2_)	enabling discoloration and enhancing both mechanical and FR performances	[128]
PP + 40 wt% Mg(OH)_2_ + 5 wt% clay (montmorillonite + sepiolite)	enhancing dispersion so mechanical performances reducing the magnesium hydroxide amount	[129]
PP + 20 wt% kenaf fiber + 20 wt% Mg(OH)_2_	enhancing mechanical performances reducing the magnesium hydroxide amount	[130]
PP + 50 wt% flax fiber + 30 wt% Mg(OH)_2_	enhancing mechanical performances reducing the magnesium hydroxide amount via natural additives	[131]
PP + 40–50 wt% wood flour + 20–30 wt% Mg(OH)_2_	enhancing mechanical performances reducing the magnesium hydroxide amount via natural additives	[132]

**Table 5:** Applications of MDH in different polymer and biopolymer matrices.

Polymer Matrix	Purpose of the Works	References
**HDPE**	Development of WPCs and compounds for flame-retardant extruded profiles (decking, boards, panels) and cable jackets/ties with low smoke.	[119,120]
**LDPE/LLDPE**	Formulations for flexible halogen-free cable sheathing, films and protective membranes for flame-retardant building applications.	[121,122,123,124,125]
**PP**	Flame-retardant WPCs for decking and structural profiles, technical molded parts (covers, ducts, housings) used in fire-risk environments.	[126,127,128,129,130,131,132]
**PVC**	Halogen-free or low-halogen cable jackets with reduced smoke (partly replacing Al(OH)_3_), rigid profiles for windows and trunking with improved fire resistance.	[117]
**PS**	Sheets and insulation panels with reduced flammability, interior decorative elements requiring low flame spread.	[135,136]
**HIPS**	Formulation of HIPS/Mg(OH)_2_ and HIPS/Mg(OH)_2_ modified with triblock copolymer styrene/butadiene/styrene materials with medium smoke suppression properties and can be used to produce nearly all elements of rolling stock equipment.	[137,138]
**PET**	Flame-retardant fibers and films for technical textiles, straps and tapes, and panels from recycled bottles for construction uses.	[139,140,141]
**POM**	Precision components (gears, guides, fittings) with improved flame resistance in electrical and mechanical devices.	[142,143,144]
**ABS**	Halogen-free housings for electrical/electronic equipment and interior automotive parts, extruded profiles and moldings targeting UL 94 V-0/V-1 ratings. Compatible with 3D-printing lavoration.	[145,146,147,148,149]
**PU**	Rigid and flexible foams for thermal insulation and cushioning with reduced burning rate, protective coatings for wood and metal.	[150,151,152,153,154]
**PA**	Technical textiles, electrical components and structural parts (connectors, supports) with enhanced self-extinguishing behavior in halogen-free systems.	[155,156,157,158,159,160]
**SEBS**	Thermoplastic elastomers for cable jackets, sleeves and soft flame-retardant coverings, seals and expansion joints with fire protection.	[161,162]
**EVA**	Thermoplastic for sheathing cable applications having improved flame fire resistance, processability and photo-oxidation behavior.	[61]
**PLA**	Bio-based flame-retardant composites for interior panels, rigid packaging prototypes and 3D-printed parts with reduced flame spread.	[163,164,165,166]
**PBS**	Biodegradable flame-retardant films and for technical packaging, temporary construction elements (formworks, spacers) with lower flammability.	[167,168]

**Table 6:** Main mechanical properties of different polymer and biopolymer matrices containing MDH at high loading.

Polymer Matrix	Stiffness/Elastic Modulus (E)	Elongation at Break (EB)	Tensile Strength (TS)	Impact Resistance	Hardness	Density	Creep Resistance	Heat Distortion Resistance	Melt Flow/Processability	References
**Polyolefins (PE; PP; EVA; PVC)**	↑ strongly	↓ moderately to strongly	↓ dramatically	↓	↑	↑ significantly	↑	↑ somewhat	↓	[61,117,119,120,121,122,123,124,125,126,127,128,129,130,131,132]
**Polystyrene (PS; HIPS)**	↑ strongly	↓ drastically	↓ moderately to strongly	↓ strongly	↑	↑ significantly	N.D.	↑ somewhat to drastically	↓ dramatically	[135,136,137,138]
**Polyamides (PA6, PA6,6)**	↑ strongly	↓ drastically	↓ moderately	↓ strongly	↑	↑ significantly	↑	↑	↓ dramatically	[155,156,157,158,159,160]
**Polyuretanes (PU)**	↑ strongly	↓ drastically	↓	↓	↑	↑ significantly	↑	N.D.	↓ dramatically	[150,151,152,153,154]
**POM**	↑ strongly	↓ drastically	↓	N.D.	↑	↑ significantly	N.D.	↑ drastically	↓ dramatically	[142,143,144]
**Rubbers (SEBS)**	↑ drastically	↓ drastically	↓ strongly	N.D.	↓ drastically	↑	N.D.	N.D.	↓ significantly	[161]
**Copolymer (ABS)**	↑ very strongly	↓ moderately to strongly	↓ moderately	↓	↑	↑	↑	↑ somewhat	↓ moderately to significantly	[145,146,147,148,149]
**Biopolymers (PLA; PBS)**	↑ very strongly	↓ drastically	↓ moderately	N.D.	↑	↑	↑ strongly	↑ somewhat	↓	[163,164,165,166,167,168]

## Data Availability

No new data were created or analyzed in this study.

## Abbreviations

The following abbreviations are used in this manuscript:

MDH	Magnesium Di-Hydroxide
MH	Magnesium Hydroxide
HDPE	High-Density Polyethene
LDPE	Low-Density Polyethene
LLDPE	Linear Low-Density Polyethene
PP	Polypropylene
PVC	Polyvinyl Chloride
PS	Polystyrene
POM	Polyoxymethylene
ABS	Acrylonitrile–Butadiene–Styrene
PU	Polyurethane
PA	Polyamide
SEBS	Styrene Ethylene Butylene Styrene
PLA	Polylactic Acid
PBS	Poly(Butylene Succinate)
VTES	Vinyl Triethoxy Silane
MA	Maleic Anhydride
PET	Poly(Ethylene Terephthalate)
HRR	Heat Release Rate
MHSH	Magnesium Hydroxide Sulfate Hydrate
NVH	Noise, Vibration, and Harshness
TA	Tannic Acid
EVA	Poly-Ethylene-Vinyl Acetate
HIPS	High-Impact Polystyrene
